# Calponin-3 deficiency augments contractile activity, plasticity, fibrogenic response and Yap/Taz transcriptional activation in lens epithelial cells and explants

**DOI:** 10.1038/s41598-020-58189-y

**Published:** 2020-01-28

**Authors:** Rupalatha Maddala, Maureen Mongan, Ying Xia, Ponugoti Vasantha Rao

**Affiliations:** 1 Department of Ophthalmology, Duke University School of Medicine, Durham, NC 27710 USA; 2 Department of Environmental Health, University of Cincinnati College of Medicine, Cincinnati, OH 45267 USA; 3 Department of Pharmacology and Cancer Biology, Duke University School of Medicine, Durham, NC 27710 USA

**Keywords:** Immunohistochemistry, RHO signalling

## Abstract

The transparent ocular lens plays a crucial role in vision by focusing light on to the retina with loss of lens transparency leading to impairment of vision. While maintenance of epithelial phenotype is recognized to be essential for lens development and function, knowledge of the identity of different molecular mechanisms regulating lens epithelial characteristics remains incomplete. This study reports that CNN-3, the acidic isoform of calponin, an actin binding contractile protein, is expressed preferentially and abundantly relative to the basic and neutral isoforms of calponin in the ocular lens, and distributes predominantly to the epithelium in both mouse and human lenses. Expression and MEKK1-mediated threonine 288 phosphorylation of CNN-3 is induced by extracellular cues including TGF-β2 and lysophosphatidic acid. Importantly, siRNA-induced deficiency of CNN3 in lens epithelial cell cultures and explants results in actin stress fiber reorganization, stimulation of focal adhesion formation, Yap activation, increases in the levels of α-smooth muscle actin, connective tissue growth factor and fibronectin, and decreases in E-cadherin expression. These results reveal that CNN3 plays a crucial role in regulating lens epithelial contractile activity and provide supporting evidence that CNN-3 deficiency is associated with the induction of epithelial plasticity, fibrogenic activity and mechanosensitive Yap/Taz transcriptional activation.

## Introduction

The avascular transparent ocular lens plays a crucial role in vision by focusing incident light on to the retina and aberrations in lens transparency (cataract) impair vision. Cataract is a leading cause of blindness in the ageing population throughout the world, and surgical replacement of opaque or cloudy lens with artificial lenses is the only treatment option available for restoration of vision in patients^1,2^. Thus, a detailed understanding of lens growth and function would provide insights into developing medical therapies to delay lens opacification. The ocular lens is located behind the cornea and iris in the anterior chamber of eye, suspended by zonules attached to the ciliary muscle. Following its development from the surface ectoderm, the lens continues to grow throughout life, with the adult lens being composed of an epithelial monolayer covering the anterior surface and fiber cells differentiated from the equatorial epithelium constituting the bulk of the lens^3,4^. The lens is encapsulated by a thick basement membrane termed the capsule and consisting of extracellular matrix. Cuboidal epithelial cells in the adult lens maintain characteristic E-cadherin and N-cadherin-based cell-cell junctions, tight junctions and apical and basal polarity^3^. Cytoskeletal and cytoskeletal-interacting proteins including actin, myosin, spectrin, integrins, catenins, scaffolding proteins and signaling proteins regulating the actin cytoskeleton networks have been demonstrated to be required for maintenance of lens epithelial phenotype, proliferation, differentiation and survival^3,5–7^. Although dysregulation of actomyosin organization, cell adhesion and cell-cell junctions is recognized to influence lens epithelial plasticity and result in transdifferentiation of lens epithelial cells into matrix producing myofibroblasts (EMT), there are gaps in our understanding of the proteins that interact with and regulate the organization and contractile characteristics of actin and myosin^8–12^.

Calponin is a well-characterized actin, myosin, tropomyosin and calcium/calmodulin binding contractile protein that inhibits actin-dependent activity of myosin Mg^2+^ ATPase^13,14^. Calponin exists as three isoforms (basic, CNN1; neutral, CNN2 and acidic, CNN3) encoded by independent genes^13^. These isoforms exhibit a molecular mass ranging between 33 to 37 kDa and share a conserved N-terminal calponin homology domain, actin binding regulatory domain, repeat region and C-terminal variable region^13^. The C-terminal domain of CNN-3 is longer than those of CNN1 and CNN2^13^. Calponin isoforms reveal cell-type and tissue-specific distribution; with CNN1 being expressed only in smooth muscle tissues while CNN2 and CNN3 are expressed in both smooth muscle and non-muscle tissues, and CNN3 being expressed abundantly in the brain tissues^13,15,16^. CNN3 is recognized as a downstream target gene of Otx2 transcription factor which is required for eye development^17^. Interestingly, while CNN1 and CNN2 null mice grow and breed normally^13^, CNN3 null mice display embryonic and neonatal lethality with severe defects in brain development^15^, indicating distinct roles for different calponin isoforms despite their shared biological roles in actin cytoskeletal networks, signaling and contraction^13^. Calponins are known to be serine/threonine phosphorylated by various kinases including protein kinase C, Ca^2+^/calmodulin kinase II, Rho kinase (CNN3) and MEKK1 (CNN3), with phosphorylation being reported to decrease actin interaction and suppression of the inhibitory effect of calponin on myosin Mg^2+^ATPase activity^13,18–22^. CNN3 has been shown to be involved in trophoblast cell fusion, wound healing and neural tube morphogenesis largely through its role in actin cytoskeletal organization^15,22,23^. A definitive role has also been recently demonstrated for CNN3 in regulating contractile activity and force generation in different types of cells^21,24^.

Our recent cDNA microarray and RNA-seq based analysis of gene expression profiles of neonatal and adult mouse lenses revealed relatively high level expression of acidic calponin (CNN3) with little to no expression of basic (CNN1) and neutral (CNN2) calponin isoforms^25^. Intrigued by this observation, we initiated studies to understand the significance of what appears to be tissue-specific expression profile of CNN-3 in the lens, and to evaluate a possible role for CNN3 in lens function. To this end, we evaluated the distribution pattern of CNN3 and found that this protein localizes predominantly to the lens epithelium, co-localizing with F-actin. Knockdown of CNN3 expression using siRNA in lens epithelial cultures and explants led to reorganization of actin stress fibers, increase in focal adhesions and epithelial transdifferentiation, and enhanced Yap/Taz transcriptional activity, revealing an important role for CNN3 in maintaining the lens epithelial phenotype.

## Results

### Preferential and abundant expression of the acidic calponin isoform (CNN3) in mouse lens, and CNN3 localization to the lens epithelium

**Figure 1:**
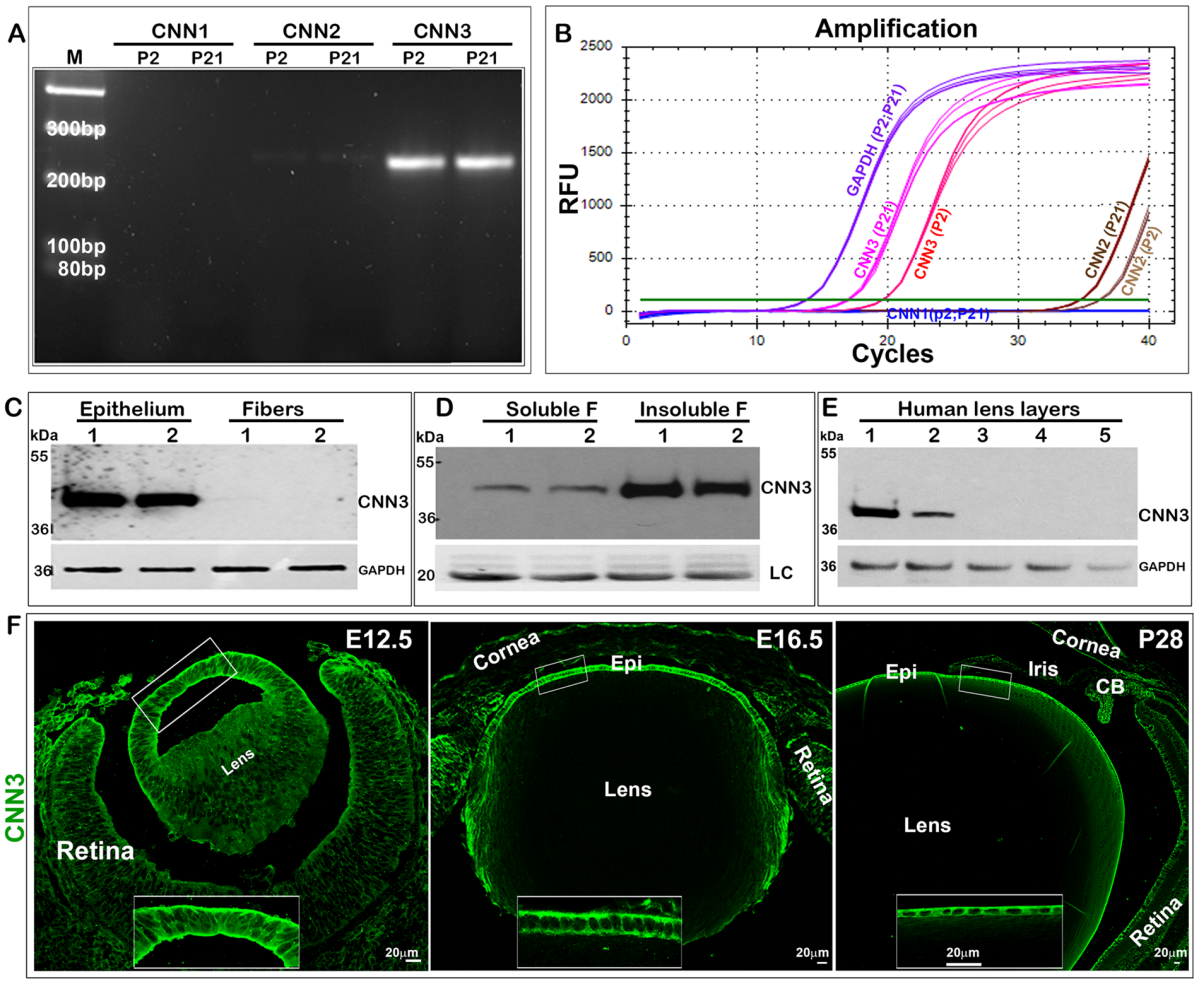
Preferential expression and discrete distribution of acidic calponin (CNN3) to the epithelium in mouse lenses. Evaluation of relative levels of expression of calponin isoforms (CNN1, CNN2 and CNN3) by RT-PCR (**A**) and qRT-PCR **(B**) analyses in P2 and P21 mouse lenses reveals preferential expression of the CNN3 isoform. (**C**) Representative immunoblot showing detection of CNN3 in the lens epithelium but not in fibers based on analysis of two independent samples. (**D**) Relative abundance of CNN3 in the insoluble/membrane fraction compared to the soluble fraction from mouse lens based on immunoblotting analysis. LC; Loading control. (**E**) Immunoblotting analysis of microdissected human lens fractions detects CNN3 predominantly in the epithelial fraction (1), with some present in the outer cortical fraction (2) but none evident in the inner cortical (3), outer nuclear (4) or central nuclear (5) fractions. GAPDH was immunoblotted as a loading control. (**F**) Immunofluorescence distribution analysis of developing (E12.5 and E16.5) and mature (P28) mouse lenses reveals preferential localization of CNN3 to the anterior lens vesicle in E12.5 and to the lens epithelium in E16.5 and P28 specimens. Inserts show magnified images of the indicated regions. Scale bars indicate image magnification.

Our laboratory has had a longstanding interest in understanding the role of actin cytoskeletal organization and cell adhesive mechanisms in lens development, growth and function^7,9,26,27^. In recent cDNA microarray^25^ and RNA-seq (unpublished)-based analyses of the mouse lens transcriptome profile we discovered that the actin, myosin, tropomyosin and calcium/calmodulin binding contractile protein CNN3, was expressed at high levels in the mouse lens relative to CNN1 and CNN2. While CNN1 expression was not detectable in these lenses, CNN2 was found to be expressed at levels that less than one percent of that of CNN3 in both neonatal and adult mouse lenses. Based on RNA-seq data (from two independent samples; unpublished), the expression level of CNN3 is ranked at 103 of a total of ~15,000 genes expressed in one month old mouse lenses, indicating a relatively high level of expression of the CNN3 gene relative to the total transcriptome profile. The cDNA microarray and RNA-seq-based observations have been confirmed by RT-PCR and q-RT-PCR analysis of neonatal (P2) and P21 mouse lenses as shown in Fig. 1A,B, respectively. Consistent with the cDNA microarray and RNA-seq data, CNN1 was not detected while CNN2 was barely detectable relative to the robust expression of CNN3 in P2 and P21 mouse lenses (Fig. 1A,B).

To characterize the distribution of CNN3 in mouse lens we then performed immunoblotting and immunofluorescence analyses using a polyclonal antibody directed against CNN3. Immunoblotting analysis of lens epithelial and fiber mass protein fractions (10 μg) derived from P23 mice detected CNN3 primarily in the epithelial fraction with little to none evident in the fiber mass fraction (Fig. 1C; shows data for two independent samples). CNN3 levels were found to be abundant in the lens membrane fraction relative to the soluble fraction (Fig. 1D). Further, based on immunofluorescence analysis of embryonic 12.5, 16.5 and P28 mouse lenses, CNN3 was found to localize predominantly and intensely to the epithelium (Fig. 1F). Even in the early developing lens (E12.5), CNN3 was noted to localize preferentially to the anterior lens vesicle that eventually becomes the lens epithelium (Fig. 1F). In addition to its presence in the epithelium, CNN3 was also seen in early elongating and differentiating secondary fibers in E16.5 lenses, being detected only at the transition zone of the lens but not in the maturing or differentiated fibers (Fig. 1F). Within the lens epithelium, CNN3 was noted to exhibit a predominantly cytosolic distribution with some localization to the lateral membrane, and to basal and apical regions of the cell body. Inserts in Fig. 1F show the magnified images of the boxed areas. Human lenses (obtained from a 56 year-old donor) were also analyzed by immunoblotting for the expression and distribution profiles of CNN3 using lysates (800 × g supernatants) derived from microdissected layers of the lens. As shown in Fig. 1E, CNN3 was detected mainly in fraction 1 (lens capsule containing the epithelium), with some being evident in fraction 2 (outer cortical fibers) but none detected in fractions from the inner cortical (fraction 3), outer nuclear (fraction 4) and central nuclear regions (fraction 5). It is possible that the human lens fraction 2 includes some epithelial contamination, since the samples were prepared from cadaver lenses received 24 hours following enucleation.

### Colocalization with F-actin and distribution of CNN3 to the primary cilium of the lens epithelial cultures and explants, respectively

**Figure 2:**
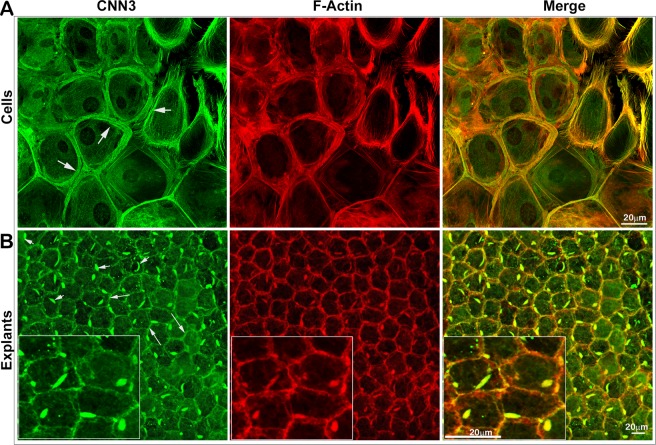
Distribution of CNN3 to the actin stress fibers, lateral membranes and primary cilia in mouse lens epithelial cells and explants. (**A**) CNN3 exhibits a stress-fiber like intense cortical distribution (green immunofluorescence; arrows), colocalizing with F-actin (red TRITC-phalloidin staining) in mouse lens epithelial primary cell cultures. (**B**) In mouse lens epithelial explants, CNN3 distributes (green immunofluorescence) to the epithelial lateral membranes (long arrows) and primary cilia (short arrows) where it colocalizes with F-actin (red TRITC-phalloidin staining). Inserts show magnified images of a small region of the same specimen. Merged images are used to indicate colocalization between CNN3 and F-actin. Scale bars indicate image magnification.

**Figure 3:**
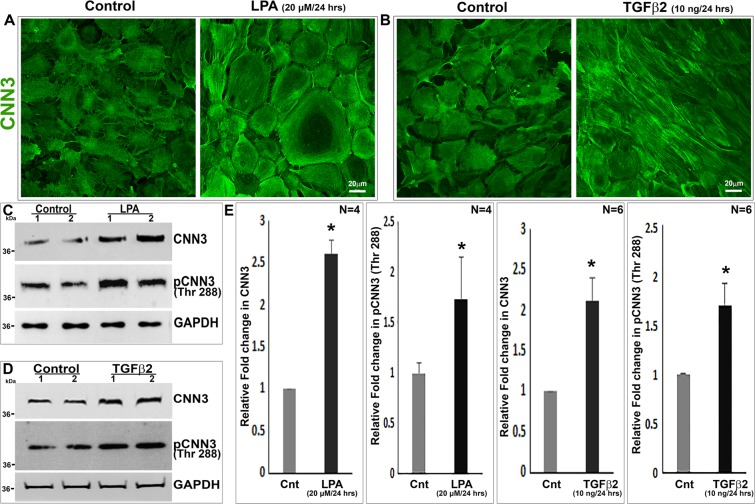
LPA and TGF-β2 induced expression and phosphorylation of CNN3 in mouse lens epithelial cultures. Serum starved mouse lens epithelial cells stimulated either with LPA (20 μM; Panel A) or TGF-β2 (10 ng/ml; Panel B) for 24 hours exhibit a contractile cell morphology with increased CNN3 immunofluorescence and its stress fiber like organization compared to untreated control cells. Moreover, there was a significant increase (*P < 0.01; n = 4; Student t test) in the protein levels of CNN3 and Thr288 phosphorylated CNN3 in both LPA and TGF-β2 (Panels C–E) treated samples compared to control cells based on immunoblotting analysis and densitometric quantification. Data are shown for two individual representative samples in immunoblots (**C,D**). GAPDH was immunoblotted as a loading control. Scale bars indicate image magnification.

We also examined the distribution of CNN3 in cultured primary lens epithelial cells (derived from the postnatal mouse lenses and passaged 2–6 times and maintained in DMEM medium containing 10% fetal bovine serum) and freshly isolated mouse lens epithelial explants (derived from P21 mouse lens) by immunofluorescence analysis. In primary lens epithelial cells, CNN3 was found to exhibit a distribution pattern very similar to that of actin stress fibers and to colocalize with F-actin filaments (labelled with TRITC-phalloidin, Fig. 2A). In these cultured epithelial cells, CNN3 immunofluorescence staining distributes intensely to the cell cortical region (Fig. 2A, arrows). In contrast, CNN3 localizes predominantly to the cell lateral membrane and primary cilium in lens epithelial explants (Fig. 2B), colocalizing with F-actin at the lateral membrane (long arrows) and in the primary cilium (Fig. 2B; short arrows; inserts show magnified images). Immunoblot analyses confirmed readily detectable CNN3 protein in mouse lens epithelial cell cultures (Fig. 3).

### LPA and TGF-β2 increase CNN3 protein levels and CNN3 Threonine 288 phosphorylation in mouse lens epithelial cell cultures

For this, we used lens epithelial primary cultures derived from P21 mice (passage of 2–6). Cells cultured on gelatin coated (2%) glass coverslips or plastic plates were serum starved for 24 hours, stimulated with lysophosphatidic acid (LPA) (20 μM) or TGF-β2 (10 ng/ml) for 24 hours prior to fixation or use in cell lysate preparation, respectively. Both LPA and TGF-β2 treated lens epithelial cells exhibited a contractile morphology with firm and stiffer cell borders compared to the relaxed morphology of control cells (Fig. 3A,B). The change in cell morphology of LPA and TGF-β2 treated cells was associated with increased immunofluorescence for CNN3 and its stress fiber- like distribution. Consistent with these observations, CNN3 protein levels were increased significantly (P < 0.01; n = 4) following a 24-hour period of stimulation of lens epithelial cells with LPA or TGF-β2 as evidenced by immunoblotting analysis (Fig. 3C–E). Immunoblotting data were normalized relative to the levels of the house keeping protein glyceraldehyde 3-phosphate dehydrogenase (GAPDH).

**Figure 4:**
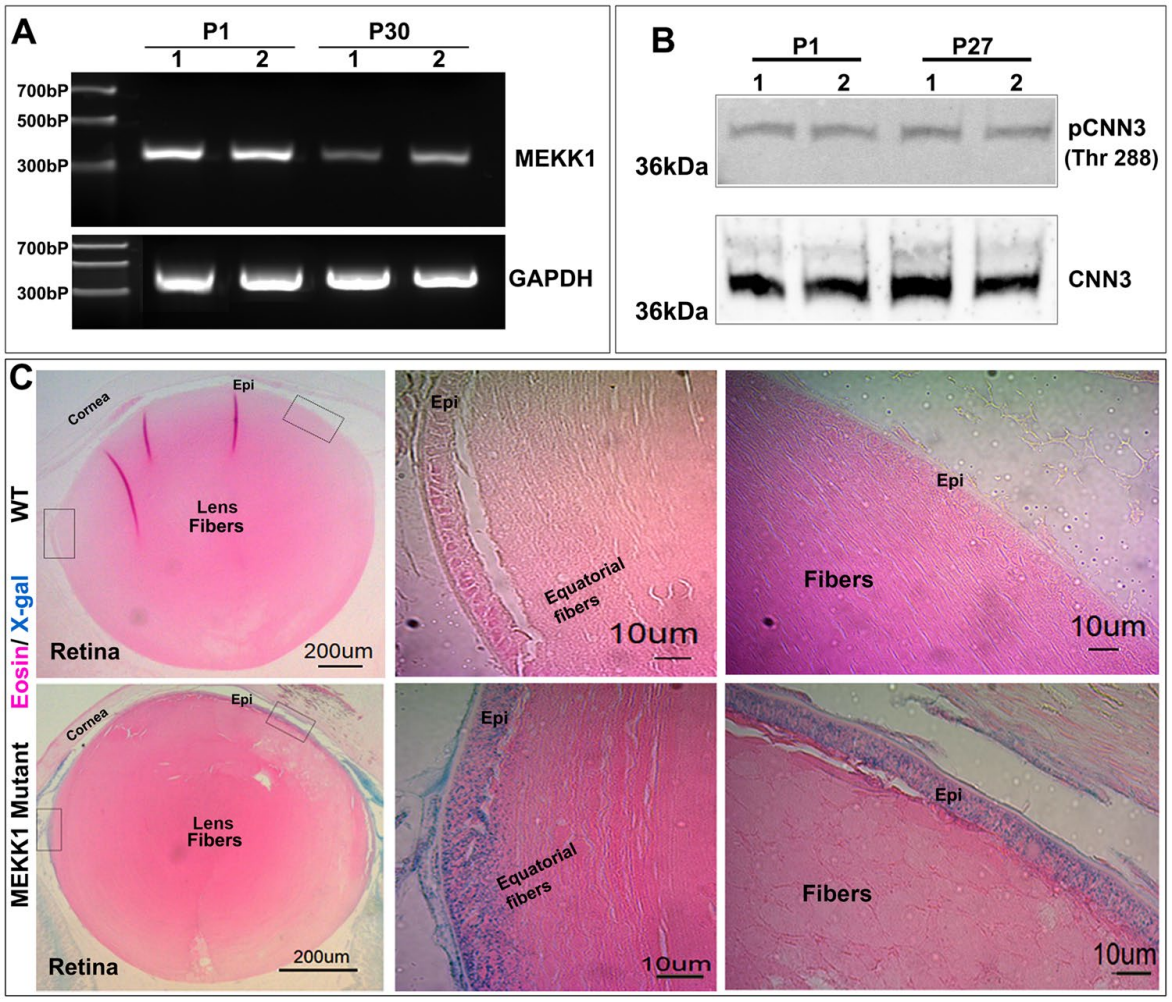
Expression and distribution of MEKK1 in mouse lens and detection of Thr288 phosphorylated CNN3 in lens epithelium. (**A**) RT-PCR based demonstration of MEKK1 expression in two individual samples of P1 and P30 mouse lenses. GAPDH was amplified in the same samples to confirm normalization of cDNA in both samples analyzed. (**B**) Immunoblot based detection of Thr288 phosphorylated CNN3 and CNN3 in two individual P1 and P27 mouse lens capsule/epithelial samples (10 µg protein). (**C**) Similar to the distribution profile observed for CNN3 (Fig. 1F), MEKK1 was found to be distributing discretely to the lens epithelium based on detection of β-galactosidase activity (blue stain, using X-gal substrate) in P7 MEKK1 mutant and wild type mice as described in the Methods section. Magnified images of the boxed areas in the left panels are shown in the middle and right panels. β-galactosidase activity (blue) was detected only in MEKK1 mutant mouse lens specimens but not in wild type samples. The eye specimens were counter stained with Eosin (red). Scale bars indicate image magnification.

Having found that both LPA and TGF- β2 induce CNN3 expression and contractile morphology in lens epithelial cells and in light of the knowledge that LPA and TGF-β2 are present in the aqueous humor and therefore accessible to the lens epithelium^28–30^, we examined whether CNN3 threonine (Thr) 288 phosphorylation status is altered by LPA and TGF-β2 treatment of mouse lens epithelial cells. We focused on assessing this attribute since CNN3 Thr288 phosphorylation has not only been shown to be regulated by MEKK1 (Mitogen-Activated Protein Kinase Kinase Kinase1) which is known to be responsive to LPA and TGF- β2 stimulation in various cell types, but to also induce contractile activity^21,31–33^. As shown in Fig. 3C,D, both LPA and TGF- β2 significantly (P < 0.01) increased the levels of CNN3 Thr288 phosphorylation relative to control cells based on immunoblot analysis using a polyclonal antibody specific to Thr288 phosphorylated CNN3 (Fig. 3E). We also confirmed the detection of Thr288 phosphorylated CNN3 in the lens epithelial explant lysates (10 µg protein, two independent samples) derived from P1 and P27 mice by immunoblot analysis (Fig. 4).

### MEKK1 exhibits a discrete distribution to the mouse lens epithelium

Since CNN3 Thr288 phosphorylation has been shown to be regulated by MEKK1,^21^ we examined the expression and distribution profile of MEKK1 in the mouse lens. After confirming expression of MEKK1 by RT-PCR analysis using RNA derived from P1 and P30 mouse lenses (Fig. 4A), we evaluated the distribution of MEKK1 protein in mouse lens using a well-characterized mouse model which expresses a catalytically inactive N-terminal domain of MEKK1 fused to β-galactosidase whose expression is regulated by a normal MEKK1 promoter. Expression and distribution of MEKK1 was analyzed in P7 mouse eyes using the β-galactosidase substrate X-Gal, as described earlier^32,34^. The *ex-vivo* detection of MEKK1-β-gal fusion protein expression in P7 mouse lens revealed positive staining (blue stain) distributing discretely to the lens epithelium but not to fiber cells (Fig. 4C). In contrast, P7 control mouse lenses did not exhibit positive staining for β-galactosidase activity in either the lens epithelium or fiber mass (Fig. 4C). These data together with those shown in Fig. 1 regarding the distribution of CNN3 in the lens imply that both CNN3 and MEKK1 localize preferentially to the epithelium in mouse lens.

### CNN3 deficiency induces contractile morphology, actin cytoskeletal reorganization and focal adhesions formation in lens epithelial cell cultures

**Figure 5:**
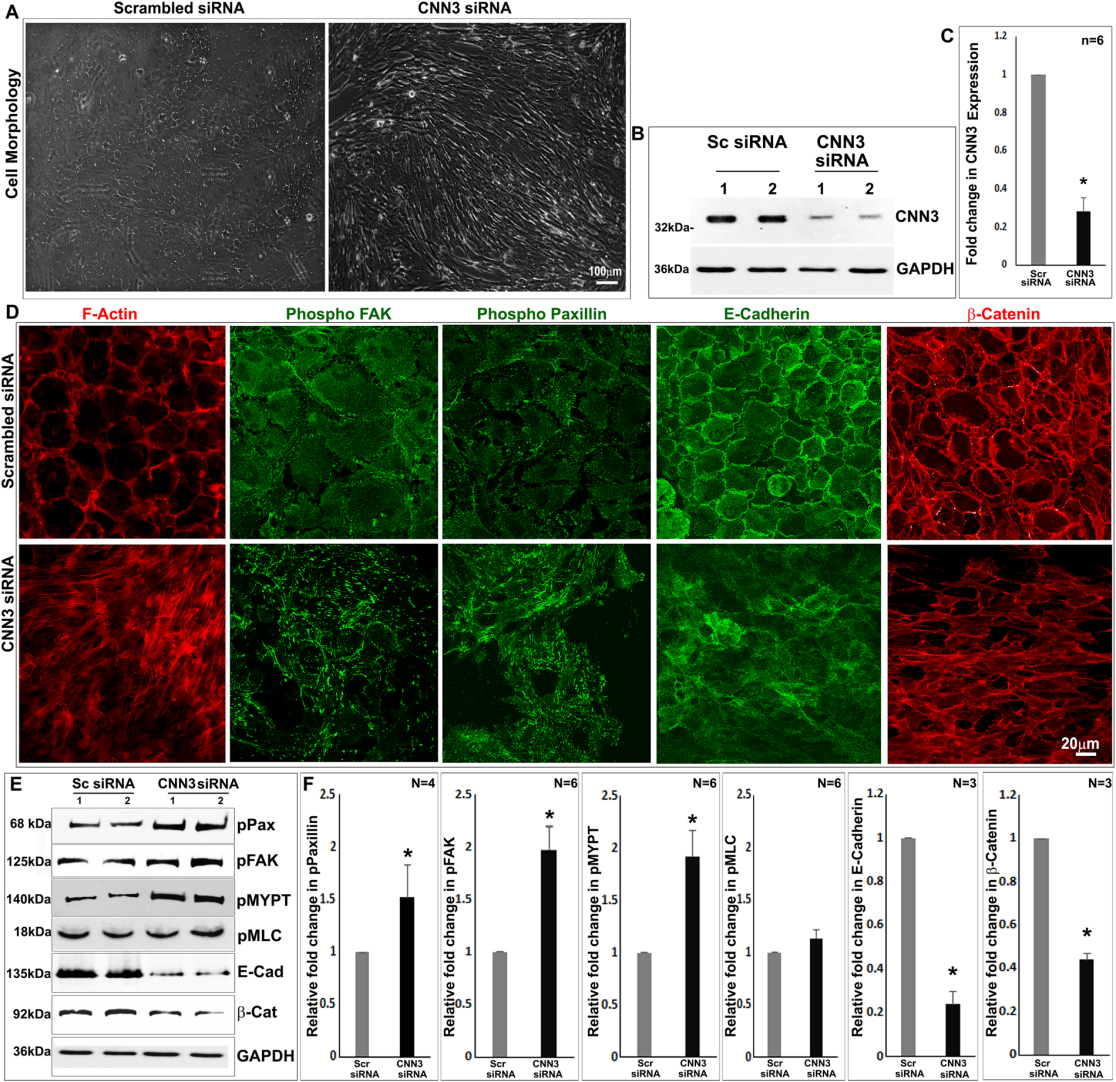
Downregulation of CNN3 expression induces changes in cell shape, reorganization of the actin cytoskeleton, increase focal adhesions and decreases in E-cadherin and β-catenin in lens epithelial cell cultures. (**A**) siRNA-mediated downregulation of CNN3 expression in serum starved mouse lens epithelial cells exhibit an altered, elongated and contractile morphology (phase contrast images) compared to control cells. (**B,C**) Downregulation of CNN3 expression in siRNA treated mouse lens epithelial cells was confirmed by a significant (*P < 0.01; Student t test) decrease in CNN3 protein levels relative to cells treated with scrambled control siRNA, as evidenced by immunoblotting analysis. (**D**) CNN3 deficient epithelial cells reveal reorganization of actin stress fibers, and increased focal adhesions formation based on increased immunofluorescence of phospho-paxillin and phospho-FAK. These cells also reveal a decrease in cell-cell adhesion based on the decrease in E-cadherin and β-catenin immunofluorescence relative to control cells. Additionally, CNN3 deficient lens epithelial cells reveal significant (*P < 0.01; Student t test) increases in the levels of p-paxillin, P-FAK and p-MYPT1 with a concomitant decrease in E-cadherin and β-catenin levels but no change in phospho-MLC relative to controls, based on immunoblotting analyses (Panel E) and densitometric quantification (Panel F). GAPDH was probed as a loading control. Sc and Scr siRNA: Scrambled siRNA. Scale bars indicate image magnification.

Because MEKK1-mediated CNN3 phosphorylation is linked to decrease actin interaction and suppression of the inhibitory effect of calponin on myosin Mg^2+^ATPase activity^13,18–22^, we evaluated the effects of siRNA-mediated suppression of CNN3 expression in lens epithelial primary cell cultures on cell shape, actin cytoskeletal organization and focal adhesions compared to cells treated with scrambled control siRNA. Lens epithelial cells treated for 48 hours with CNN3-specific siRNA and maintained under serum free conditions (24 hours) exhibited alterations in cell morphology from hexagonal to elongated fibroblast like shape compared to control cells treated with scrambled control siRNA (Fig. 5A; phase contrast images). Immunoblotting analysis of lysates derived from CNN3 siRNA treated lens epithelial cells confirmed a significant decrease (by 70%; n = 6) in the levels of CNN3 protein relative to control cells (Fig. 5B,C).

CNN3 siRNA treated lens epithelial cells exhibited a dramatic reorganization of the actin cytoskeleton, from the cortical ring like distribution observed under control conditions to a spreading and filamentous profile extending between the anterior to posterior poles throughout the cell body (Fig. 5D). This reorganization of actin filament in CNN3 deficient lens epithelial cells was associated with a robust increase in focal adhesions formation based on increased immunofluorescence staining of phospho-paxillin and phospho-focal adhesion kinase (pFAK) compared to control cells (Fig. 5D). Significant increases in protein levels of phospho-paxillin, phospho-FAK and phospho-MYPT1 (myosin phosphatase subunit) were also observed in CNN3 deficient lens epithelial cells relative to control cells (Fig. 5E,F). No differences were noted however, in the total levels of paxillin, FAK and total myosin light chain (MLC) in CNN3 deficient cells relative to control cells treated with scrambled siRNA (Supplemental Material; Fig. S1).

Although we observed prominent changes in actin cytoskeletal organization and increased focal adhesions formation together with phosphorylation of MYPT1, a known substrate of Rho kinase in CNN3 deficient lens epithelial cells, there were no significant changes in the levels of phosphorylated MLC and total MLC in these cells based on immunoblotting analysis (Figs. 5E,F and S1). Further, CNN3 deficiency was associated with decreased immunostaining for E-cadherin and β-catenin. Consistent with these results, we also observed a significant decrease in the levels of both E-cadherin and β-catenin in CNN3 deficient relative to control lens epithelial cells (Fig. 5E,F).

### CNN3 deficiency induces Yap/Taz transcriptional activity in lens epithelial cells

**Figure 6:**
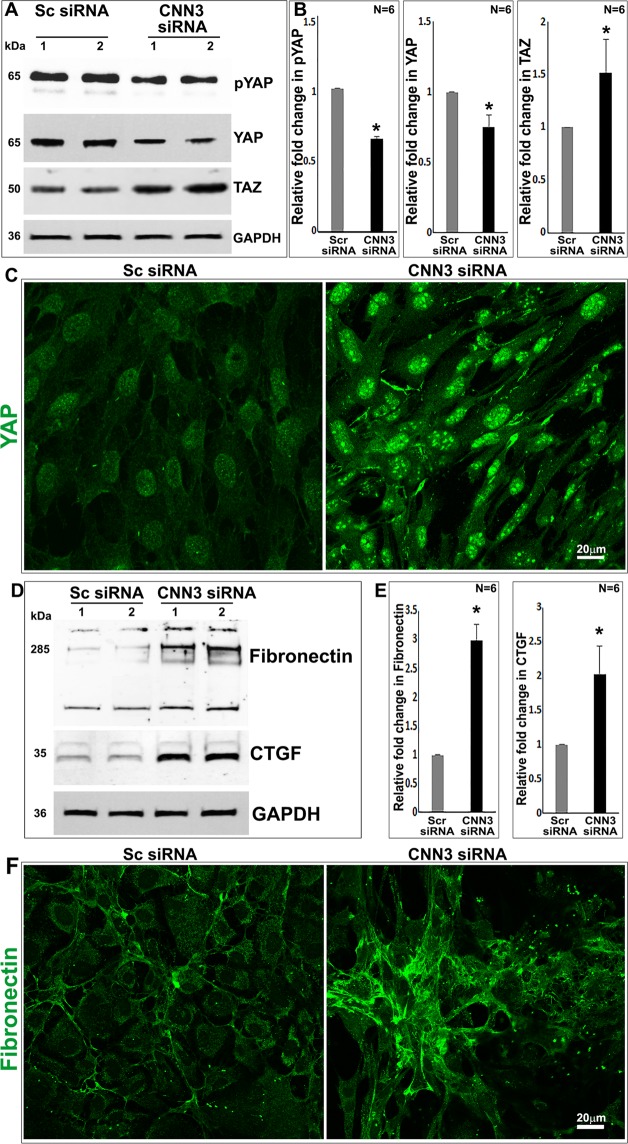
CNN3 deficiency induces activation of the Yap/Taz and fibrogenic responses in lens epithelial cell cultures. siRNA-mediated downregulation of CNN3 expression in mouse lens epithelial cells as described in the Methods section results in significant decreases (*P < 0.01; Student t test) in the levels of phospho-Yap and total Yap, together with significant increase (*P < 0.01; Student t test) in the protein levels of Taz compared to controls cells treated with scrambled siRNA, based on immunoblotting analysis (panel A) and densitometric quantification (panel B). There was robust nuclear translocation of Yap in CNN3 deficient lens epithelial cells based on immunofluorescence analysis (panel C). Significant increases (*P < 0.05; Student t test) were also observed in the levels of CTGF and fibronectin (immunoblotting, panel D and densitometry, panel E) and fibronectin production (immunofluorescence; panel F) in CNN3 deficient lens epithelial cells compared to respective control cells. Scale bars indicate image magnification and GAPDH was immunoblotted for loading control. Sc and Scr siRNA: Scrambled siRNA.

Since CNN3 deficiency was noted to trigger alterations in cellular contractile morphology and actin cytoskeletal organization, and formation of focal adhesions in lens epithelial cells, all of which collectively indicate an increase in cell tension, we asked whether these changes impact mechanical force-linked transcriptional mechanisms in lens epithelial cells. To address this question, we analyzed the activation status of Yap (Yes-associated protein) and Taz (transcriptional coactivator with a PDZ-binding domain), two closely related transcriptional coactivator proteins recognized to play a key role in integrating mechanical cues with regulation of gene expression^35^. CNN3 deficient lens epithelial cells exhibited a significant decrease in the levels of phospho-Yap and increase in the levels of Taz relative to control cells treated with a scrambled siRNA (Fig. 6A,B). Moreover, although there was a significant decrease in the levels of Yap in CNN3 deficient lens epithelial cells, there was a robust increase in nuclear translocation of Yap in these cells, indicating an activation of Yap/Taz compared to the respective control cells (Fig. 6C). Based on the results suggesting activation of Yap/Taz, we evaluated whether there were corresponding changes in the expression of Yap/Taz target gene- connective tissue growth factor (CTGF) and fibronectin, by immunoblotting analysis of CNN3 deficient and control lens epithelial cells. The levels of both CTGF and fibronectin were significantly increased in CNN3 deficient lens cells compared to control cells (Fig. 6D,E). The fibronectin immunoblot also showed additional immunopositive bands other than the prominent 280 kDa protein which appear to be the aggregated and fragmented products of fibronectin (Fig. 6D). The changes in fibronectin expression under CNN3 deficiency in lens epithelial cells were further validated by immunofluorescence staining (Fig. 6F).

### CNN3 knockdown induces epithelial plasticity and fibrogenic activity in association with Yap activation in lens epithelial explants

**Figure 7:**
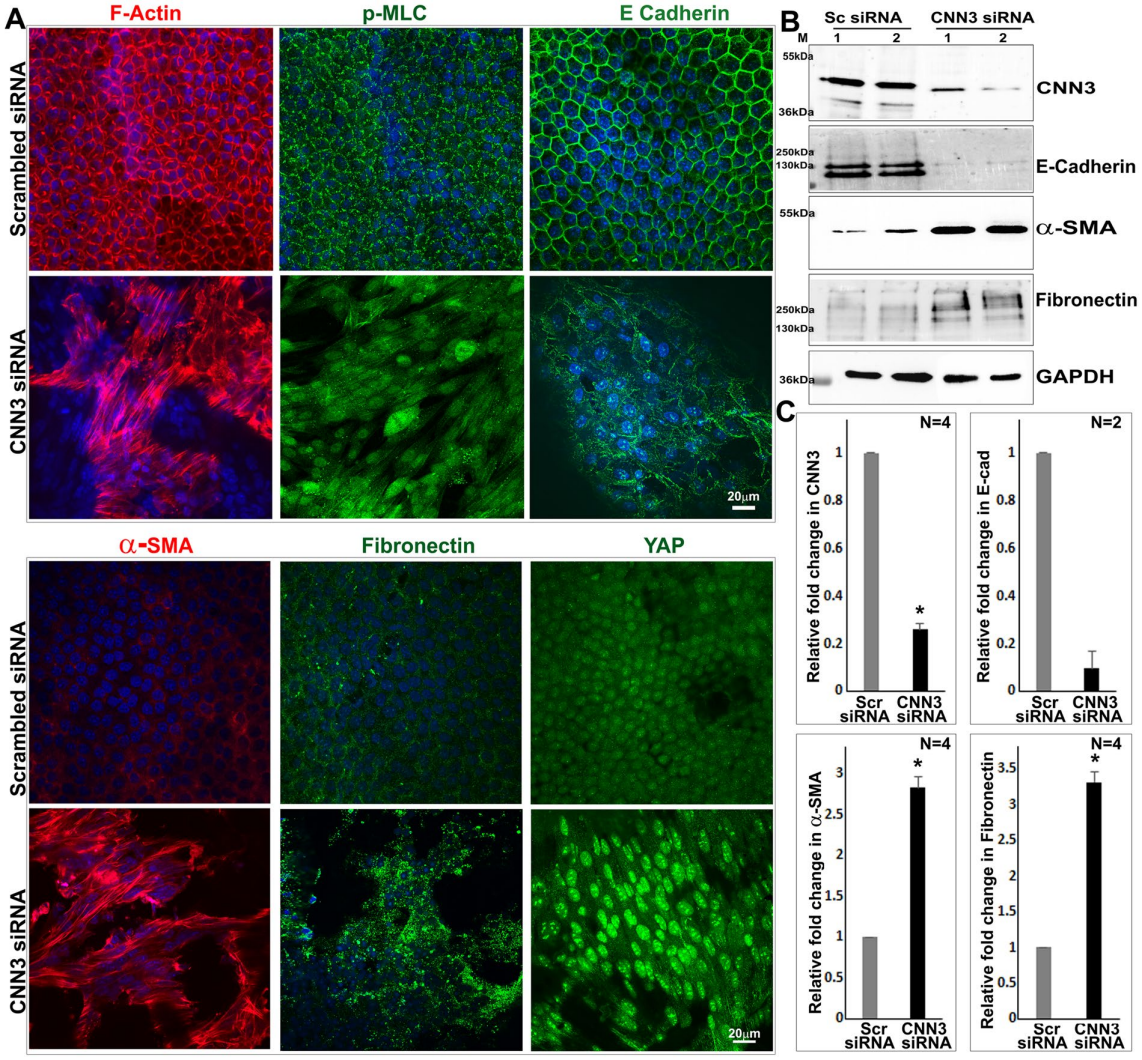
CNN3 knockdown by siRNA treatment of lens epithelial explants induces reorganization of actin stress fibers, and increases α-SMA and fibronectin in association with increased Yap nuclear translocation and decreased E-Cadherin. (**A**). Treatment of mouse lens epithelial explants with CNN3 siRNA for 72 hours induces changes in cell morphology from the epithelial hexagonal shape to an elongated morphology with reorganization of actin stress fibers (TRITC-phalloidin staining) distributing transversely throughout the cell body, reorganization of p-MLC similar to actin stress fibers, a robust increase in α-SMA and fibronectin levels and translocation of Yap into the nucleus and decrease in E-cadherin staining compared to cells treated with a scrambled siRNA as evidenced by immunofluorescence evaluation. Scale bars indicate image magnification. (**B,C**) Immunoblot based quantification under the above described conditions showed a significant decrease (*P < 0.05; n = 4; Student t test) in CNN3 and ~90% (n = 2) decrease in E-cadherin protein levels, and an increase in the levels of α-SMA and fibronectin (n = 4; *P < 0.05; Student t test) in CNN3 siRNA treated epithelial explants compared to the respective controls. GAPDH was immunoblotted as a loading control. Data for two representative samples (Lanes 1 and 2) are provided in panel B. Sc and Scr siRNA: Scrambled siRNA.

The effect of siRNA-mediated CNN3 deficiency was also evaluated using lens epithelial explants derived from postnatal mice (P21). Treatment with CNN3 siRNA for 72 hours in the absence of serum consistently led to curling of lens epithelial explants compared to epithelial explants treated with scrambled control siRNA, presumably due to increased contractile activity. The appearance of folds and wrinkles in CNN3 deficient explants interfered with a thorough imaging analysis of these specimens. Immunoblotting analysis was used to confirm downregulation of CNN3 protein levels (by 74%; n = 4) in lens epithelial explants treated with CNN3-specific siRNA relative to control explants treated with scrambled siRNA (Fig. 7B,C). There was a dramatic reorganization of actin cytoskeleton and phosphorylated MLC in CNN3 deficient lens epithelial explants (Fig. 7A). CNN3 deficient lens explants also showed an obvious reduction in E-cadherin immunofluorescence in association with increased α-SMA and fibronectin immunofluorescence staining compared to control specimens (Fig. 7A). Similar to F-actin organization, αSMA also exhibits stress fiber like distribution in the CNN3 deficient explants. In control specimens, there was very little αSMA and fibronectin immunofluorescence staining (Fig. 7A). Most significantly, immunofluorescence analysis confirmed robust translocation of Yap into the epithelial cell nucleus in CNN3 deficient lens explants, relative to the respective controls (Fig. 7A). A significant increase was also documented in the levels of αSMA and fibronectin (n = 4) and decrease in E-cadherin (n = 2; by 90%) in CNN3 deficient lens epithelial explants compared to control specimens (Fig. 7B,C). Collectively, these lens epithelial explant observations are consistent with those noted in lens epithelial cell cultures.

## Discussion

**Figure 8:**
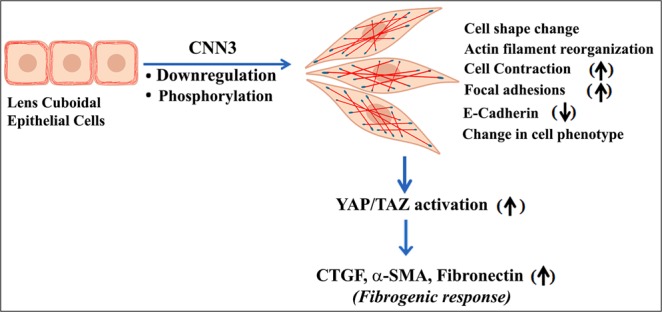
Schematic illustration of CNN3 deficiency and phosphorylation induced changes in lens epithelial cell shape in association with reorganization of actin stress fibers and increased focal adhesions and a decrease in E-cadherin-based cell-cell junctions, leading to stimulation of Yap/Taz transcriptional activity, and expression of markers of epithelial to mesenchymal transition (EMT) including accumulation of extracellular matrix. These observations collectively imply a critical role for CNN3 in maintaining the lens epithelial phenotype. Upward and downward pointing arrows indicate upregulated and downregulated responses, respectively. The red and blue stains depict organization of actin filaments and focal adhesions, respectively.

Although the actin cytoskeleton and actomyosin contractile activity have been recognized to play a crucial role in lens epithelial morphogenesis and maintenance, the identity of various proteins that interact with and regulate organization of the actin cytoskeleton and actomyosin contraction in the lens are not well characterized^5,10^. In this regard, and for the first time to the best of our knowledge, this study reports the acidic isoform of calponin (CNN3) is preferentially and abundantly expressed in the ocular lens, distributing predominantly to the lens epithelium. CNN3 colocalizes with F-actin, with the two proteins distributing to the plasma membrane in the lens epithelium. Although CNN3 expression in the lens epithelium is induced by external cues (LPA and TGF-beta2), its Thr288 phosphorylation is also up-regulated, likely mediated through MEKK1. This phosphorylation is presumed to suppressing the inhibitory effects of CNN3 and leads to the increase of the myosin Mg^2+^ATPase activity and contractility^21^. Consequently, the effects of CNN3 Thr 288 phosphorylation are expected to be similar as CNN3 deficiency. Downregulation of CNN3 expression in lens epithelial cells was found to induce changes in cell shape, reorganization of actin cytoskeleton and formation of focal adhesions resulting in activation of mechanosensitive transcription factor Yap and to stimulate the production of CTGF, αSMA and fibronectin in association with decreased E-cadherin and β-catenin in the lens epithelium. These observations reveal that both CNN3 and MEKK1-mediated phosphorylation of CNN3 play a crucial role in regulation of contractile force and maintenance of the lens epithelial phenotype. Based on these observations we speculate that dysregulation of CNN3 expression and phosphorylation induces EMT and fibrogenic activity in lens epithelial cells by the contractile mechanical force augmented activation of Yap/Taz transcriptional activity and cell plasticity (Fig. 8).

Lens epithelial morphogenesis, polarity and cell-cell junction integrity have been demonstrated to be regulated by actin cytoskeletal organization, non-muscle myosin II, integrins, ankyrin-G and Rho, Rac and Rap1 GTPases^3,6,8–12,26,36^. Additionally, disruption of lens epithelial actin cytoskeletal organization, polarity and cell-cell junctional integrity have been recognized to impair epithelial morphogenesis, induce EMT and increase production of extracellular matrix^8,9,12,37^. Interestingly, development of posterior capsular cataracts (PCO) is a very common secondary pathology in patients with intraocular lenses, and is associated with altered lens epithelial plasticity and EMT^38,39^. However, the causes underlying susceptibility of lens epithelial cells to undergo EMT and become plastic and fibrogenic are far from understood^38^. This also raises a question regarding the identity of unique molecular mechanisms governing maintenance of lens epithelial phenotype and polarity. Towards addressing these questions and having recognized the importance of various signaling pathways and proteins in regulating actin cytoskeletal organization and cell-cell junctions, we initiated an assessment of the transcriptome profile of various actin and actomyosin interacting proteins in the mouse lens based on RNA-seq and cDNA microarray analyses. In initial analyses, we recognized that CNN3 was expressed not only preferentially but abundantly in the lens relative to the CNN1 and CNN2 isoforms, and also that phosphorylation of CNN3 residue Thr288 was readily detected in lens epithelium.

Based on the above observations we proceeded to determine the cellular localization of CNN3 in both mouse lens epithelial cultures and epithelial explants. As has been shown in other cell types^21,24^, in lens epithelial cultures, CNN3 was found to colocalize with F-actin and distribute to actin stress fibers, whereas in lens epithelial explants, CNN3 was noted to distribute predominantly to the plasma membrane, and colocalize with F-actin in this region. Additionally, in the lens epithelial explant, CNN3 exhibited an intense distribution to the primary cilium which has been shown to be involved in planar cell polarity and maintaining lens polarity and alignment of fibers cells along the lens anterior to posterior axis^40,41^. Studies have demonstrated that lens fiber cells possess an apically situated cilium and polarized towards the anterior pole, and also that when lens epithelial explants are isolated, the primary cilium containing apical portion of fiber cells breaks down with the broken part retained by epithelial cells of explants, as found in this study^40^. In the lens primary cilium, CNN3 exhibits colocalization with F-actin. The actin cytoskeleton and actin cytoskeletal regulatory proteins along with microtubules are well-recognized components of the primary cilium^42^. Although we do not currently have experimental evidence, it appears quite likely that based on its distribution profile to the primary cilium, CNN3 may play a critical role in maintenance of lens epithelial and fiber cell polarity and the apical-apical interaction between these two types of cells in the lens.

Interestingly, in this study we found evidence for the ability of LPA and TGF-β2 to stimulate an increase in CNN3 levels in lens epithelial cultures, indicating that the expression of CNN3 is subject to regulation by growth factors and extracellular cues. Additionally, the contractile activity of calponin is known to be negatively regulated in part via Protein kinase C-mediated phosphorylation, and that phosphorylated calponin by protein kinase C has been demonstrated to suppress its inhibitory activity of the Mg2 + ATPase of myosin II^18,19^. Rho kinase and MEKK1 have also been reported to regulate CNN3 phosphorylation in various cell types. Although Rho kinase-mediated phosphorylation of CNN3 has been shown to increase actin stress fiber formation and regulate wound healing and trophoblast cell fusion, a definitive involvement of CNN3 in this response was not clear^22–24^. On the other hand, MEKK1 regulated phosphorylation of CNN3 residue Thr288 and decreased CNN3 expression have been demonstrated to increase contractile activity in C2C12 and U2OS cells, respectively^21,24^. In this study, we confirmed not only Thr288 phosphorylation of CNN3 in the lens epithelium, but also the induction of CNN3 phosphorylation by LPA and TGF-β2, stimuli that also known to activate MEKK1 signaling^32,34^. We further demonstrated that MEKK1 exhibits a distribution profile like that of CNN3, with both proteins localizing discretely to the lens epithelium. No other kinases have been found to mediate phosphorylation of CNN3 on amino acid residue Thr288^21^. It is also important to note that MEKK1 has been demonstrated to regulate actin stress, focal adhesions formation and phosphorylation of myosin light chain^43–46^, with CNN3 being reported to partially account for MEKK1-regulated actin cytoskeletal changes^21^.

Our results show that siRNA-mediated deficit of CNN3 expression in lens epithelial cultures and epithelial explants is associated with a reorganization of actin stress fibers together with an increase in focal adhesion formation indicating enhanced contractile activity. This observation is consistent with increased contractile activity of actin stress fibers reported in U2OS osteosarcoma cells under deficiency of CNN3^24^. More importantly, we found convincing evidence for stimulation of Yap transcriptional activity in CNN3 deficient lens epithelial cells as evidenced by decreased levels of phosphorylated Yap and increased Yap nuclear translocation, and recorded an increase in levels of CTGF, a well-recognized Yap regulated target gene^35^. Together with these observations, we also obtained evidence for a decrease in E-cadherin and β-catenin levels in CNN3 deficient lens epithelial cultures and epithelial explants. Yap/Taz transcriptional activators are recognized to be regulated by cell shape and polarity, actin cytoskeletal integrity, cell adhesion, Rho GTPases and mechanical forces^35,47,48^, while the Hippo-Yap pathway is known to play a crucial role in regulating organ size, growth and cell proliferation^35^. Therefore, it is possible that altered cell polarity, cell-cell junctions and increased contractile force generated under deficiency of CNN3 activates Yap/Taz transcriptional activity in the lens epithelium, leading to induction of CTGF expression, αSMA and fibronectin all markers of a fibrogenic response associated with EMT (Fig. 8). LPA is recognized to regulate Yap/Taz activity via inactivation of LATS1/2,^49^ a Hippo pathway kinase, and we have also demonstrated a definitive association between LPA, LPA receptor-mediated increase in contractile activity and activation of Yap/Taz and fibrotic responses in trabecular meshwork cells^50^. TGF-β has similarly been shown to mediate crosstalk between Yap/Taz transcription and Wnt signaling and induce EMT and fibrogenic response^51^. Although deficiency of CNN3 is reported to induce neuronal stem cell proliferation via increased nuclear translocation of β-catenin and Wnt activation^15^, we did not detect nuclear translocation of β-catenin in lens epithelial cells and explants.

In conclusion, this study indicates an important role for CNN3 in regulating the lens epithelial phenotype and reveals that CNN3 deficiency induces changes in cell shape, cell adhesion, polarity and an increase in contractile activity leading to Yap/Taz activation and EMT. In future experiments it is necessary to investigate the role of CNN3 in lens development and function by developing a conditional knockout model and to decipher regulation of CNN3 expression and phosphorylation in the context of EMT, which is considered to play a crucial role in PCO^38^.

## Methods

### Mice

All studies using mice (male and female animals of the C57BL/6J strain; MEKK1 mutant and CD1 strain) were carried out in accordance with the recommendations of the Guide for the Care and Use of Laboratory Animals of the National Institutes of Health. The protocol was approved by the Institutional Animal Care and Use Committee (IACUC) of the Duke University School of Medicine.

### Cell culture and treatments

Primary epithelial cells were isolated by collagenase IV digestion of lens capsule/epithelium derived from postnatal (P21 to P30) mice. Briefly, mouse lenses were microdissected using a dissecting microscope to isolate the capsules/epithelium, cut into tiny pieces and digested for 90 minutes at 37 °C in medium 199 (Gibco) containing 1.5 mg/ml collagenase IV and 0.2 mg/ml albumin. The digested contents were centrifuged (at 2500 rpm) for 10 minutes at 37 °C, and the cell pellet was suspended in Dulbecco’s modified Eagle’s medium (DMEM) containing 10% fetal bovine serum (FBS), and plated on gelatin (2%) coated petri plates. Cells derived using this protocol and passages between 2–6 times were used for immunoblotting and immunofluorescence analyses. Confluent cell cultures grown either on 2% gelatin coated glass coverslips or plastic petri plates as described above were serum starved for 24 hours prior to being treated with LPA (20 μm; Cayman chemicals) or TGF-β2 (10 ng/ml; Millipore-Sigma) for 24 hours. For siRNA treatment, lens epithelial cells grown on gelatin coated glass coverslips or plastic petri plates (80% confluent) as described above were treated with Opti-MEM (Gibco) containing Lipofectamine® RNAiMAX transfection reagent (ThermoFisher Scientific) and 50 pmoles of CNN3 siRNA or scrambled siRNA (Santa Cruz Biotechnologies) for 30 minutes prior to supplementation with media containing 10% FBS and continued incubation for 48 hours. Cells were then incubated for another 24 hours in serum free medium prior to use in immunostaining or immunoblotting analyses.

### Lens epithelial explants and treatments

To isolate lens epithelial explants, mouse (P21) lenses were dissected and rinsed. After carefully making a small nick at the posterior side of the capsule, 4 to 5 flaps of capsule were peeled away as described by Sugiyama *et al*.^40^. These epithelial explants were either fixed immediately with 4% buffered paraformaldehyde for immunostaining or treated with CNN3 siRNA as described above for lens epithelial cell cultures. Following siRNA treatment, explants were fixed for immunofluorescence analysis or used to prepare lysates for immunoblot analysis.

### RT-PCR and q-RT-PCR

Expression profiles of calponin isoforms and MEKK1 in mouse lens tissue was initially performed by RT-PCR (Reverse transcription polymerase chain reaction) analysis of total RNA extracted from mouse lenses (P2, and P21-day-old) using the RNeasy Micro kit (Qiagen). The Advantage RT-for-PCR Kit (Clontech) was used to synthesize first-strand cDNA from total RNA isolated. First strand cDNA and the Advantage® 2 PCR Kit (Clontech) were used to quantitate expression of calponin isoforms (CNN1, 2 & 3) and MEKK1 genes. The following mouse oligonucleotide primer sets (forward/reverse) were used in PCR reactions:

Calponin-1: 5′-GCCCAGAAATACGACCATCA-3/5′-GTACCCAGTTTGGGATCATAGAG-3′; product size (518 bp).

Calponin-2: 5′-CAGAACTCCGAAGCTGGATAG-3′/5′-CGCTCTCGAACAGGTCATT-3′; product size (249 bp).

Calponin-3: 5′-GGACTCGGAGGCATCTTTATG/5′-CCCATACACGCTCATTCCTT-3′; product size: (239 bp).

MEKK1: 5′-GTGGTGAAGCCAATCCCTATTA-3′/5′-CTGTCTCCTCCAATCAGGAAAG-3′; product size (712 bp).

PCR products were sequenced to confirm identity.

Real-time PCR (qRT-PCR) quantitation of calponin isoforms was performed using CFX96 Touch™ qPCR system (Bio-Rad). Briefly, the first strand cDNA pools from P2 and P21 were normalized to the levels of housekeeping gene GAPDH (Glyceraldehyde 3-phosphate dehydrogenase). PCR reactions were carried out in triplicate using iQ™ SYBR® Green Supermix, (Bio-Rad). The fold difference in gene expression of calponin isoforms was calculated by the comparative threshold (CT) method, as described by the manufacturer.

### Immunofluorescence and Imaging

#### Lens frozen sections

Embryonic heads (E12.5 and E16.5) and adult P28 day-old mouse eyes, were fixed in 4% buffered paraformaldehyde, cryoprotected, embedded in OCT (optical cutting temperature) compound and 10 μm sections were cut in sagittal plane using Microm HM 550 as described by us earlier^9^. Cryosections were incubated overnight with CNN3 rabbit polyclonal antibody (details are described in Table S1; Supplemental material) as we described earlier^9^. Following this, sections were washed and incubated with Alexa Fluor® 488 goat anti-rabbit secondary antibody (Invitrogen) before image capture using an Eclipse 90i confocal laser scanning microscope (Nikon Instruments, Inc.)^9^.

#### Lens epithelial explants

Lens epithelial explants treated with either CNN3 siRNA or scrambled siRNA as described above were fixed for 30 minutes with 4% buffered paraformaldehyde before being permeabilized and blocked as described for lens cryosections. Explants were then stained for F-actin using TRITC (Tetramethylrhodamine)-Phalloidin (1:500 dilution) and immunostained for p-MLC, E-Cadherin, α-SMA, fibronectin and YAP using the respective antibodies (Table S1). Images were acquired using an Eclipse 90i confocal laser scanning microscope.

#### Lens epithelial cells

Mouse lens primary epithelial cell cultures grown on glass cover slips with 10% FBS media as outlined above were fixed, permeabilized and immunostained for CNN3 alone or co-stained with TRITC-Phalloidin as described earlier^27^. Similarly, cells treated either with CNN3 siRNA or scrambled siRNA were fixed, permeabilized and immunostanied for p-FAK, p-Paxillin, E-Cadherin, β-catenin, fibronectin and YAP using respective antibodies (Table S1) in conjunction with appropriate secondary antibodies^27^. Coverslips were mounted on glass slides with Immu-mount and images were captured using an Eclipse 90i confocal laser scanning microscope.

### X-gal immunostaining

MEKK1 mutant and CD-1 wild type (Albino) postnatal (P7) eye samples were immersion fixed in 2% paraformaldehyde, 0.2% glutaraldehyde for 20 minutes at room temperature and washed in 1X PBS three times at 10 minutes each. Samples were immersed in stain solution containing 100 mM NaHPO4 (pH 7.3), 5 mM K4Fe(CN)6, 5 mM K3Fe(CN)6, 2 mM MgCl2, 0.02%NP40, 1 mg/ml X-gal and incubated overnight at 37 °C as described earlier^34^. Stained samples were immersion fixed in 4% paraformaldehyde overnight at 4 °C and processed to paraffin and sectioned at 8–14 μm. Sections were counterstained by brief immersion in diluted Eosin, and mounted with xylene mounting medium and a coverslip for imaging. Images were captured using an AxioScope.A1 (Zeiss).

### Immunoblots

#### Lens tissue and epithelial cell lysates

Wild type mouse lenses (P21 day-old) were homogenized in hypotonic buffer, separated the soluble and insoluble (membrane-enriched) fractions and protein concentration was estimated as described earlier^25^. To determine the presence of CNN3 in human lens, a 56-year-old human donor lens (Miracles In Sight, NC, USA), was microdissected into five regions (L1: capsule/epithelium, L2: outer cortical region, L3: inner cortical region, L4: outer nucleus and L5: central nucleus), homogenized in hypotonic buffer as described above. The 800 × g supernatants generated from each region was immunoblotted. Additionally, freshly extracted mouse lenses from weaned mice were microdissected to separate the capsule/epithelium and fiber cell mass using a dissecting microscope. Dissected tissue fractions were homogenized in hypotonic buffer as mentioned above, and equal amounts of protein from the 800 × g supernatants were used in immunoblotting analyses. Similarly, mouse lens epithelial explants transfected with CNN3 siRNA and scrambled siRNA were homogenized in 8 M urea buffer containing 20 mM Tris, 23 mM glycine, 10 mM dithiothreitol and saturated sucrose containing protease and phosphatase inhibitors^25^. The 800 × g supernatants derived from explants were used for immunoblotting analyses.

For the mouse lens epithelial cell cultures, cells subjected to different treatments were rinsed with cold phosphate buffered saline (PBS) and extracted with cold 10% trichloroacetic acid. The cell precipitates (derived from the centrifugation at 13,000 rpm) were washed with cold PBS and dissolved in 8 M urea buffer described above using sonication. Protein content was determined using Pierce™ 660 nm Protein Assay Reagent.

#### Immunoblot analyses

Equal amounts of protein from both treated and untreated or soluble and membrane fractions described above were resolved by SDS-PAGE (Sodium dodecyl sulphate-polyacrylamide gel electrophoresis) using gels consisting of an appropriate percent of acrylamide depending on the proteins to be analyzed. Proteins were electrophoretically transferred to nitrocellulose membrane which was then blocked with 5% milk protein in Tris buffered saline (TBS) containing 1% Tween-20 for 2 hours prior to being incubated overnight at 4 ^ο^C with the primary antibodies of interest (Table S2; Supplemental Material) in blocking buffer^25^. Membranes were washed with TBS buffer containing 1% Tween 20 and incubated with appropriate secondary antibodies for 2 hours at room temperature. Immunoblots were developed by enhanced chemiluminescence (Millipore Sigma, St. Louis, MO), followed by scanning and analysis using ChemiDoc Touch imaging and Image Lab™ Touch Software (Bio-Rad Laboratories, Hercules, Ca), respectively^25^. Densitometry analyses were carried out using Image J software. For determining the distribution of CNN3 between the soluble and insoluble (membrane enriched) fractions of the mouse lens described in Fig. 1, protein loading was normalized using a common protein band indicated in Fig. 1 as loading control (LC). For this, equal amounts of protein (10 µg) from both the soluble and insoluble fractions were separated on SDS-PAGE and separated proteins stained with Gelcode blue stain. A protein band which exhibited equal distribution between the two samples was used as a loading control, and staining intensity was assed using Image J.

### Statistical analyses

All data are reported as mean ± SEM (standard error of the mean) values based upon analysis of at least 4 independent samples, until otherwise mentioned. Comparisons between groups were performed using the Student’s t-test, with values of *P < 0.05 being considered statistically significant.

## Supplementary information

Supplementary data.

Supplement for original Western blots and Gels.
