# Supplementary material for: Calponin-3 deficiency augments contractile activity, plasticity, fibrogenic response and Yap/Taz transcriptional activation in lens epithelial cells and explants
Source: Sci Rep. 2020 Jan 28;10:1295. doi: 10.1038/s41598-020-58189-y (PMC6987178; doi:10.1038/s41598-020-58189-y)
Supplement: Supplementary file 1 — Supplementary data. [file 41598_2020_58189_MOESM1_ESM.pdf]

Calponin-3 deficiency augments contractile activity, plasticity, fibrogenic response and Yap/Taz transcriptional activation in lens epithelial cells and explants

Rupalatha Maddala,[1] Maureen Mongan,[2] Ying Xia[2] and Ponugoti Vasantha Rao[1, 3*]

Table S1: Antibodies used in immunofluorescence staining

Antibodies	Cat. No.	Source	Dilution	Used for
Calponin 3, Rabbit polyclonal Ab	sc-16724	Santa Cruz Biotechnology, Dallas, TX	1:500	Cryosections, explants and cell cultures.
Phalloidin–Tetramethylrhodamine B isothiocyanate (TRITC)	P1951	Millipore Sigma, and St. Louis, MO	1:500	Explants and cell cultures.
Phospho-FAK (Tyr397) Rabbit polyclonal mAb	700255	Thermo Fisher Scientific, Waltham, MA.	1:200	Cell cultures.
E-Cadherin (24E10) Rabbit mAb	3195	Cell Signaling Technologies, Inc. Danvers, MA.	1:200	Explants and cell cultures.
Phospho-Paxillin (Tyr118) Rabbit polyclonal Ab	2541	Cell Signaling Technologies, Inc.Danvers, MA	1:200	Cell cultures.
β-Catenin (D10A8) Rabbit mAb	8480	Cell Signaling Technologies, Inc, Danvers, MA	1:200	Cell cultures.
YAP Rabbit polyclonal Ab	4912	Cell Signaling Technologies, Inc., Danvers, MA	1:200	Explants and cell cultures.
Fibronectin Rabbit polyclonal Ab		Harold P. Erickson, PhD, Cell Biology, Duke University, Durham, NC, USA	1:200	Explants and cell cultures.
Phospho-Myosin Light Chain 2 (Thr18/Ser19) Rabbit polyclonal Ab	3674	Cell Signaling Technologies, Inc., Danvers, MA	1:200	Explants
α-smooth muscle actin- Cy3 conjugated	C6198	Millipore Sigma, and St. Louis, MO	1:200	Explants
Alexa Fluor 488, Goat anti-Rabbit IgG	A11008	Thermo Fisher Scientific, Waltham, MA.	1:500	Cryosections, explants and cell cultures.
Alexa Fluor 568, Goat anti-Rabbit IgG	A11004	Thermo Fisher Scientific, Waltham, MA.	1:500	Explants and cell cultures.
Hoechst 33258, Pentahydrate	H21491	Thermo Fisher Scientific, Waltham, MA.	1:500	Cell cultures.

Table S2: Antibodies used in immunoblotting analyses

Antibodies	Cat. No.	Source	Dilution
Calponin 3, Rabbit polyclonal Ab	sc-16724	Santa Cruz Biotechnology, Dallas, TX	1:1000
Phospho-Calponin 3 (Thr288) Rabbit polyclonal Ab		Yasuhiro Sawada, M.D., Ph.D from National Rehabilitation Center for Persons with Disabilities, Japan	1:5000
Phospho-FAK (Tyr397) Rabbit mAb	700255	Thermo Fisher Scientific, Waltham, MA.	1:2000
E-Cadherin (24E10) Rabbit mAb	3195	Cell Signaling Technologies, Inc. Danvers, MA.	1:1000
Phospho-Paxillin (Tyr118) Rabbit polyclonal Ab	2541	Cell Signaling Technologies, Inc. Danvers, MA	1:1000
β-Catenin (D10A8) Rabbit mAb	8480	Cell Signaling Technologies, Inc., Danvers, MA	1:1000
Phospho-MYPT1 (Ser507) Rabbit polyclonal Ab	3040	Cell Signaling Technologies, Inc., Danvers, MA	1:1000
Phospho-YAP (Ser127) Rabbit mAb	13008	Cell Signaling Technologies, Inc., Danvers, MA	1:1000
YAP Rabbit polyclonal Ab	4912	Cell Signaling Technologies, Inc., Danvers, MA	1:1000
YAP/TAZ (D24E4) Rabbit mAb	8418	Cell Signaling Technologies, Inc., Danvers, MA	1:1000
Fibronectin Rabbit polyclonal Ab		Harold P. Erickson, PhD, Cell Biology, Duke University, Durham, NC, USA.	1:8000
CTGF Rabbit polyclonal Ab	ab6992	Abcam, Cambridge, MA	1: 1000
α-smooth muscle actin- monoclonal Ab	A2547	Millipore Sigma, and St. Louis, MO	1:1000
Phospho-Myosin Light Chain 2 (Thr18/Ser19) Rabbit polyclonal Ab	3674	Cell Signaling Technologies, Inc., Danvers, MA	1:1000
GAPDH monoclonal Ab	60004-1	Protein tech Group, Chicago, IL	1:5000

Fig. S1

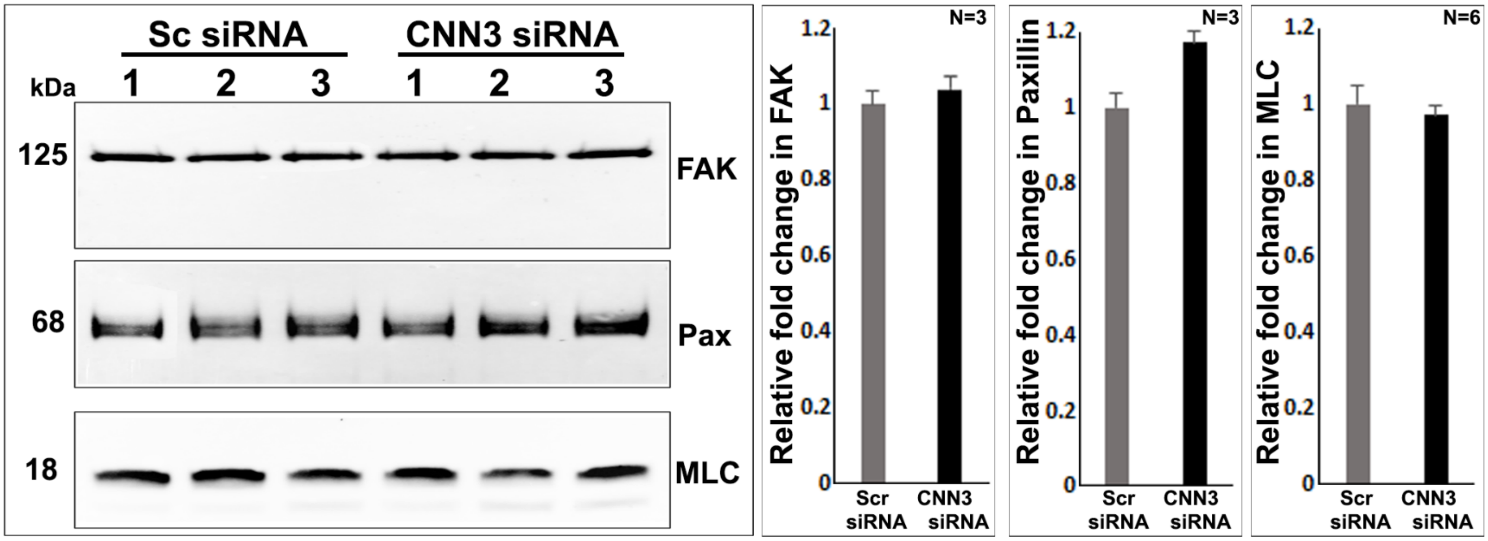

Fig. S1. Levels of total myosin light chain, paxillin and focal adhesion kinase in CNN3 deficient mouse lens epithelial cells. Having found changes in the levels of phosphorylated paxillin and FAK in lens epithelial cells under deficiency of CNN3 (Fig. 5), we determined the levels of total paxillin, FAK and MLC by immunoblotting and densitometry based quantification. This analysis revealed no difference in the levels of total paxillin, FAK and MLC between the CNN3 deficient (CNN3 siRNA treated) and control (scrambled siRNA treated) cells. Immunoblots are shown for three (lanes 1-3) representative biological replicates. Sc and Scr siRNA: Scrambled siRNA.
