# Supplementary material for: Calponin-3 deficiency augments contractile activity, plasticity, fibrogenic response and Yap/Taz transcriptional activation in lens epithelial cells and explants
Source: Sci Rep. 2020 Jan 28;10:1295. doi: 10.1038/s41598-020-58189-y (PMC6987178; doi:10.1038/s41598-020-58189-y)

Calponin-3 deficiency augments contractile activity, plasticity, fibrogenic response and Yap/Taz transcriptional activation in lens epithelial cells and explants

Rupalatha Maddala,[1] Maureen Mongan,[2] Ying Xia[2] and Ponugoti Vasantha Rao[1, 3*]

Original immunoblot images used in the manuscript

Fig. 1C

CNN3 in Lens Epithelium & Fibers

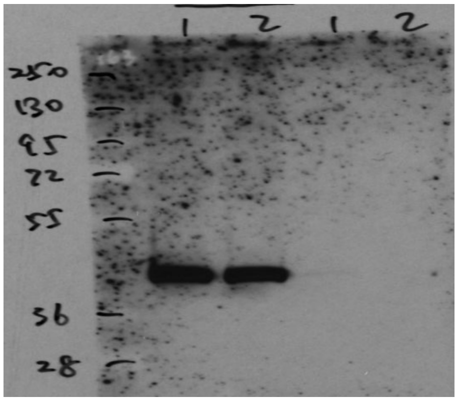

GAPDH in Lens Epithelium & Fibers

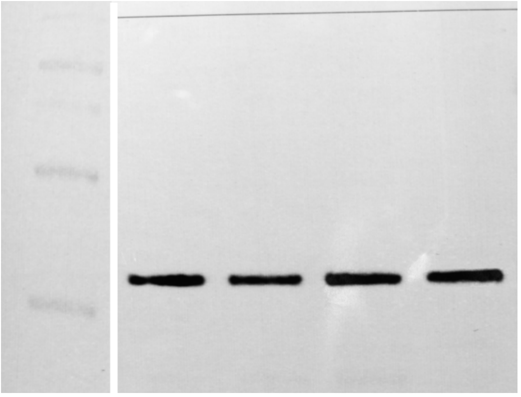

Fig. 1D

CNN3 in Lens cytosolic & membrane fraction

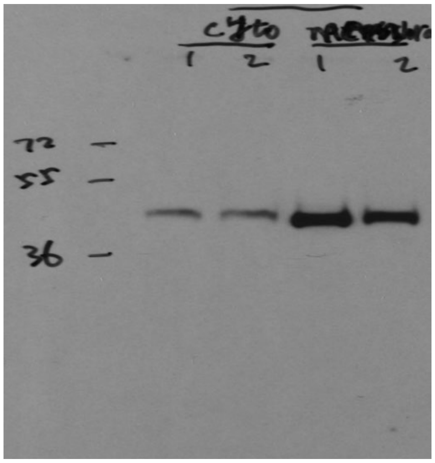

LC in Lens cytosolic & membrane fraction

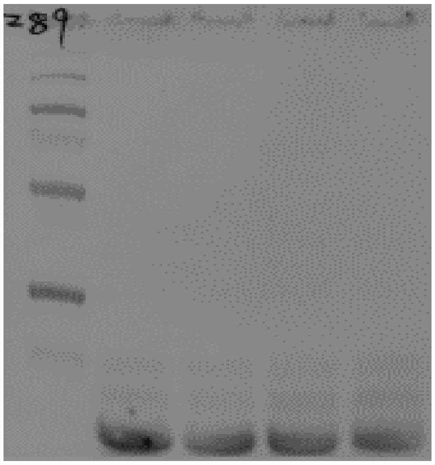

Fig. 1E

CNN3 in Human Lens layers

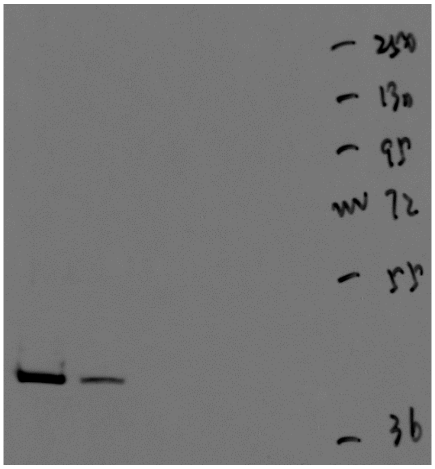

GAPDH in Human Lens layers

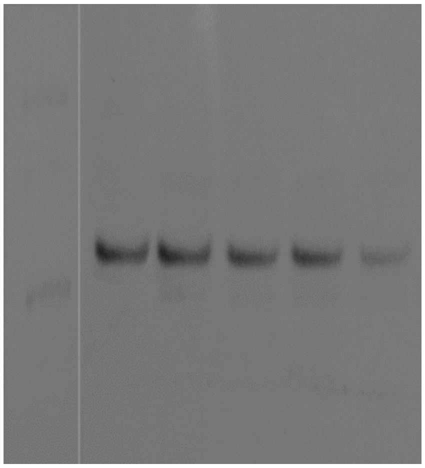

Fig. 3C

CNN3 with LPA treatment

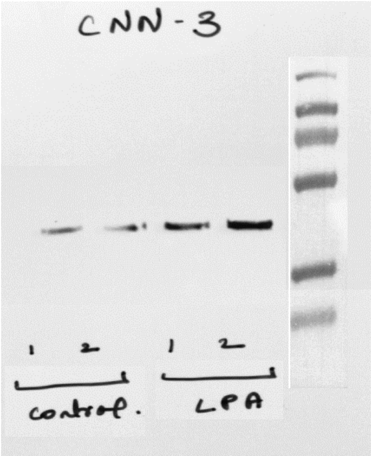

pCNN3 with LPA treatment

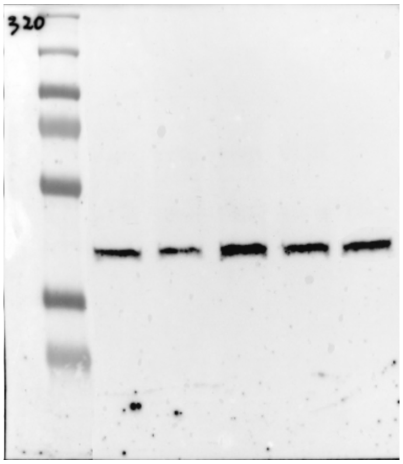

GAPDH with LPA treatment

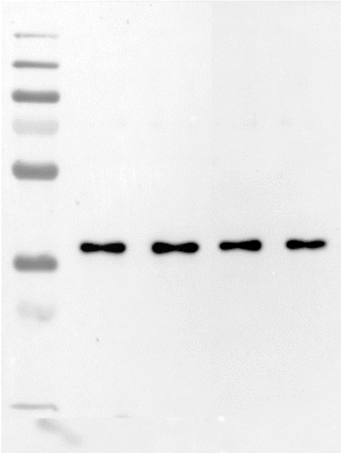

Fig. 3D

CNN3 with TGFβ2 treatment

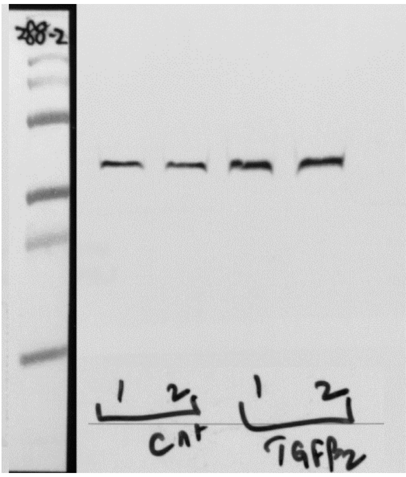

pCNN3 with TGFβ2 treatment

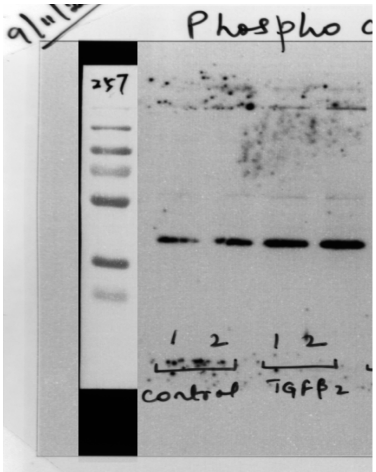

GAPDH with TGFβ2 treatment

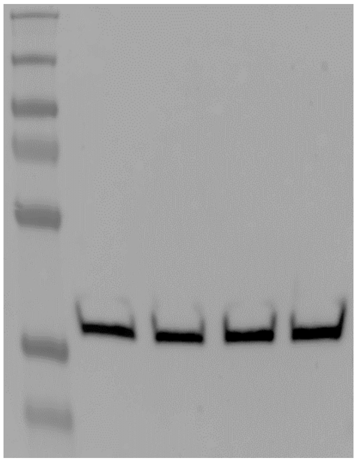

Fig. 4B

pCNN3 (Thr 288) in P1 and P27 lens epithelium

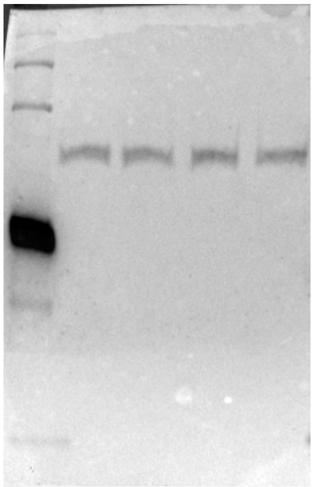

CNN3 in P1 and P27 lens epithelium

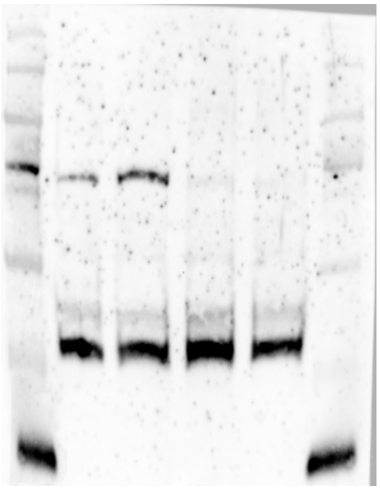

Fig. 5B

CNN3 expression in siRNA treated MLEC

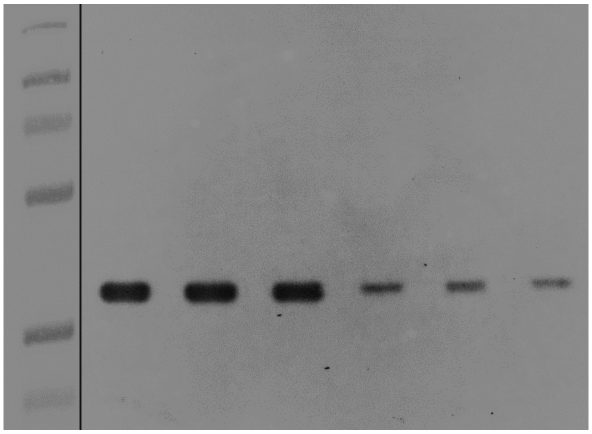

GAPDH expression in siRNA treated MLEC

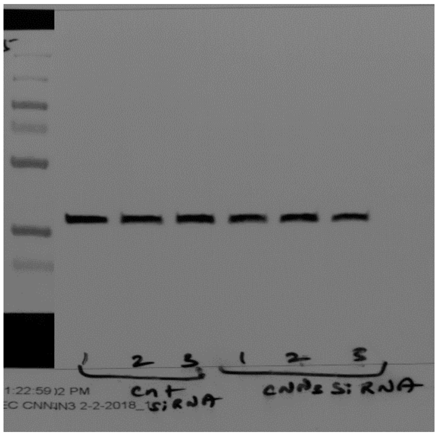

Fig. 5E

pPax expression in siRNA treated MLEC

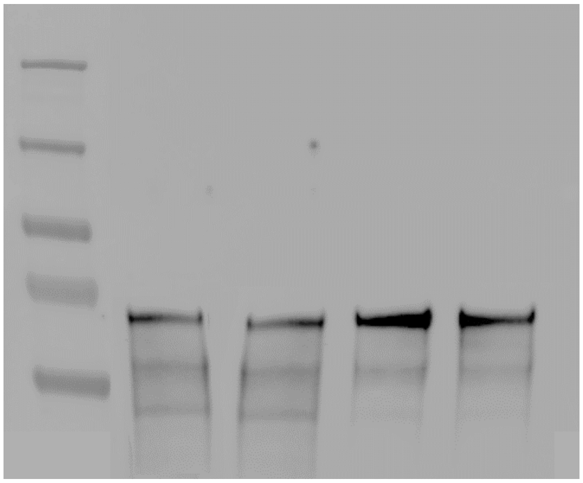

pFAK expression in siRNA treated MLEC

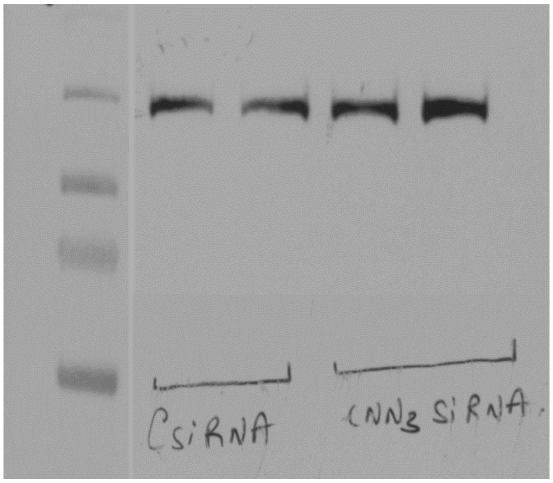

pMYPT expression in siRNA treated MLEC

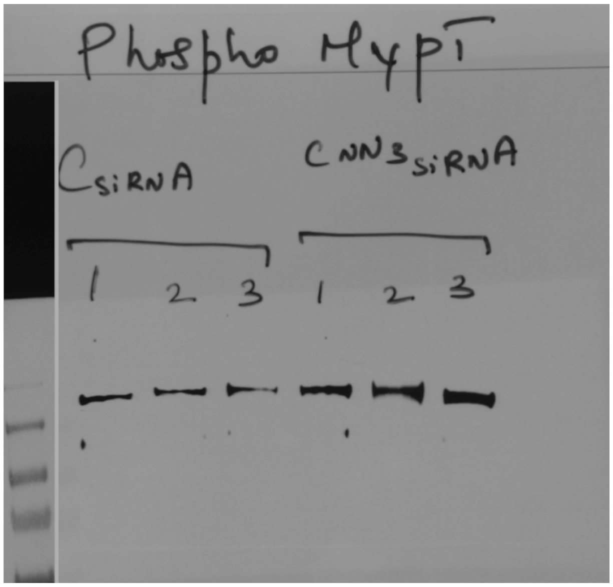

pMLC expression in siRNA treated MLEC

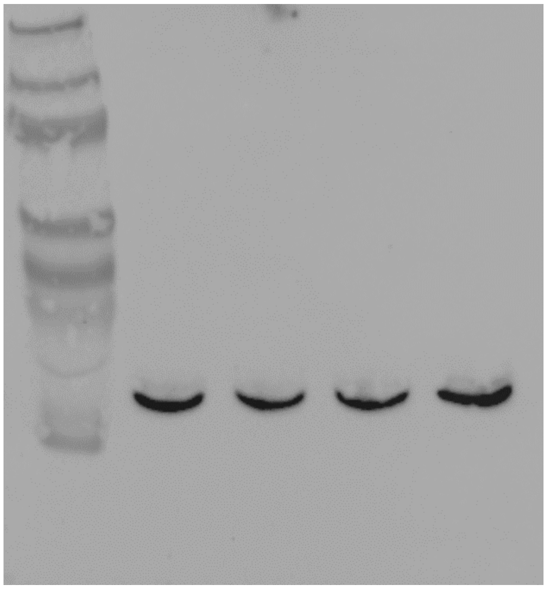

Ecad expression in siRNA treated MLEC

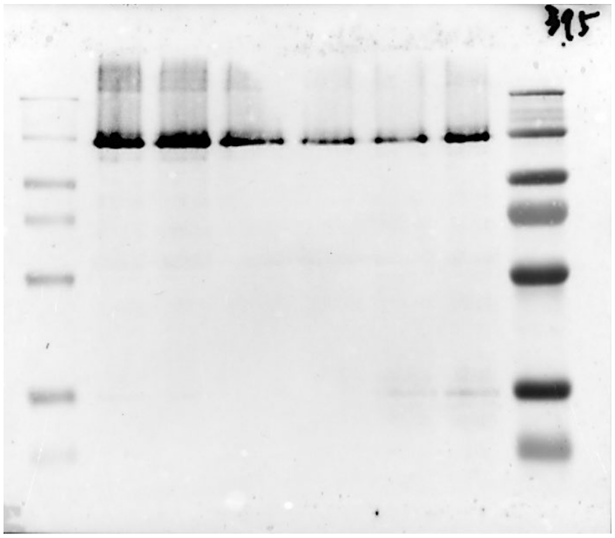

β-Catenin expression in siRNA treated MLEC

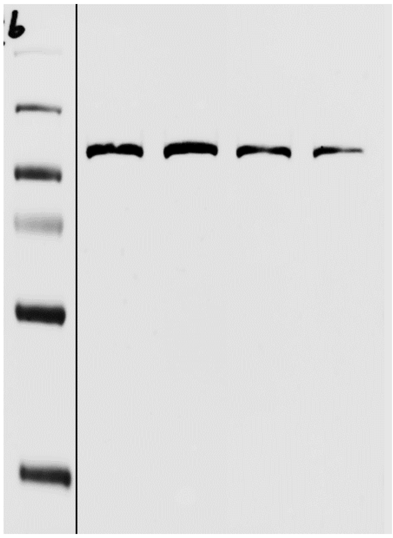

GAPDH expression in siRNA treated MLEC

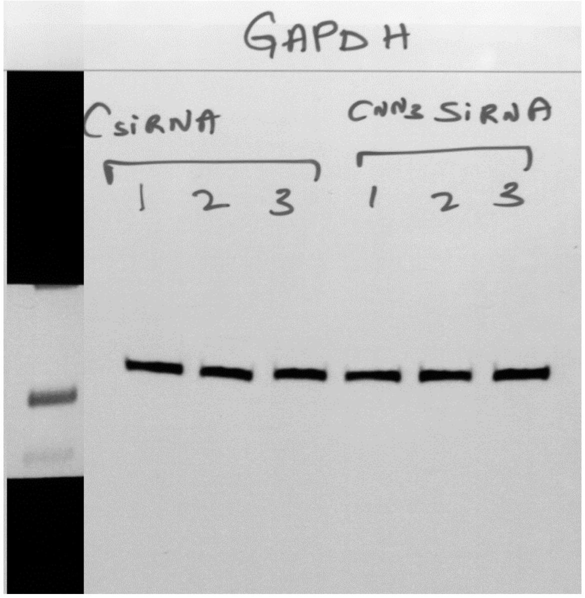

Fig. 6A

pYAP expression in siRNA treated MLEC

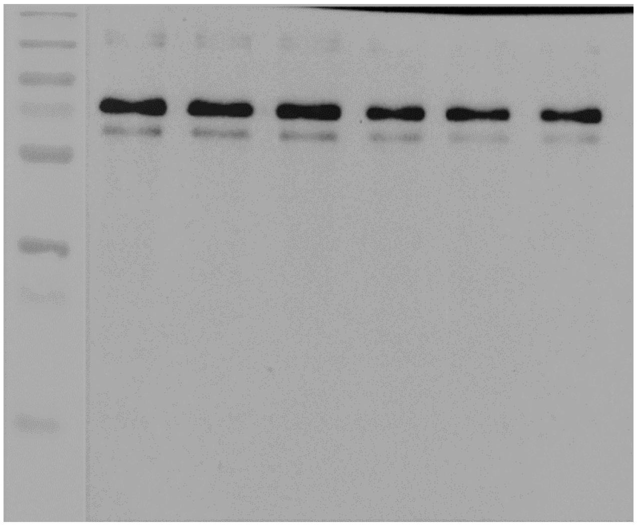

Total YAP expression in siRNA treated MLEC

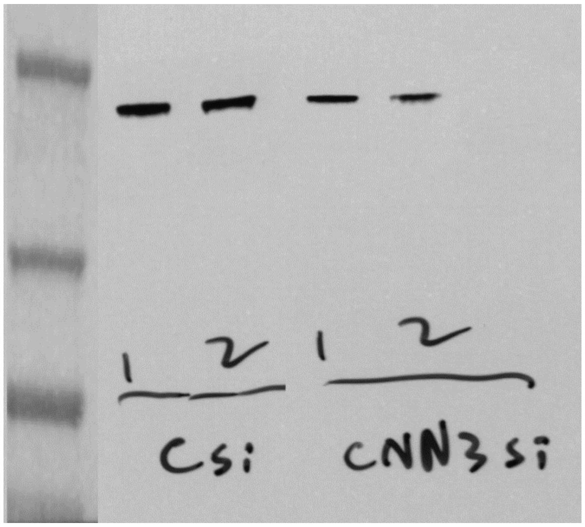

TAZ expression in siRNA treated MLEC

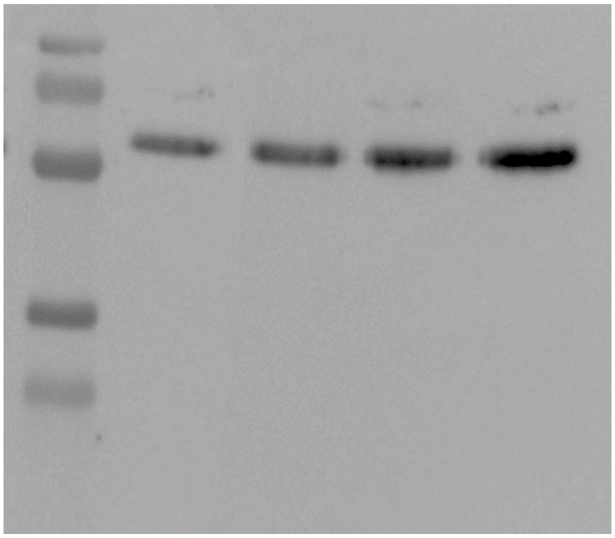

GAPDH expression in siRNA treated MLEC

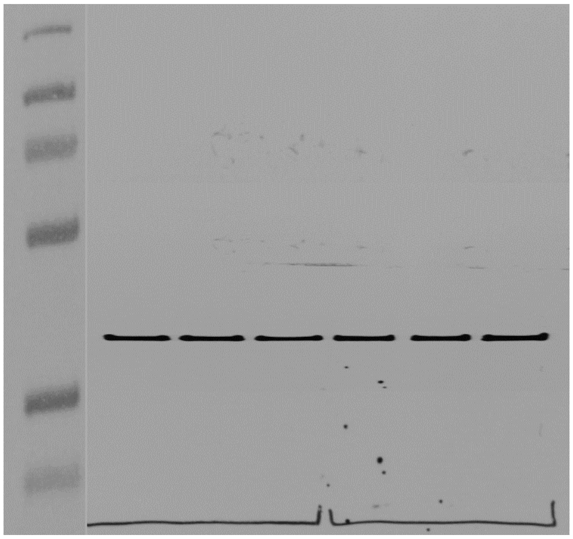

Fig. 6D

Fibronectin expression in siRNA treated MLEC

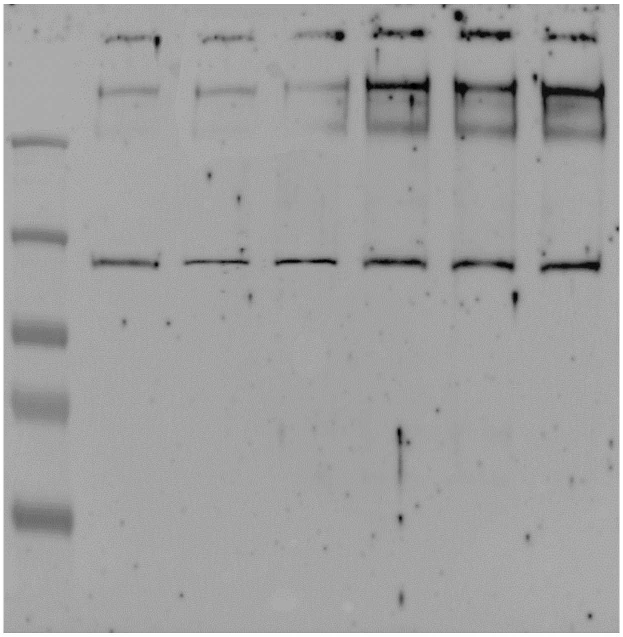

GAPDH expression in siRNA treated MLEC

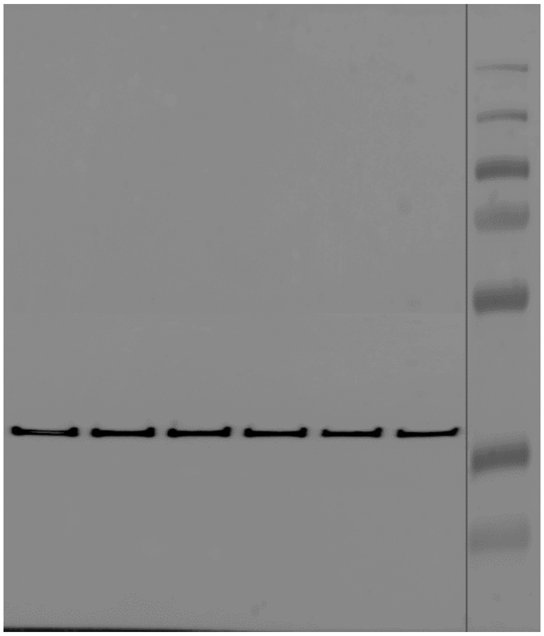

CTGF expression in siRNA treated MLEC

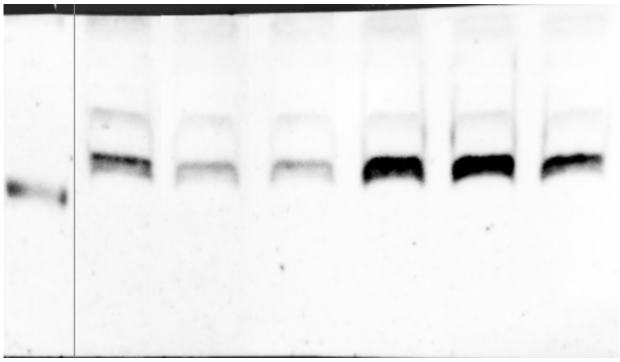

Fig. 7B

CNN3 expression in siRNA treated ML explants

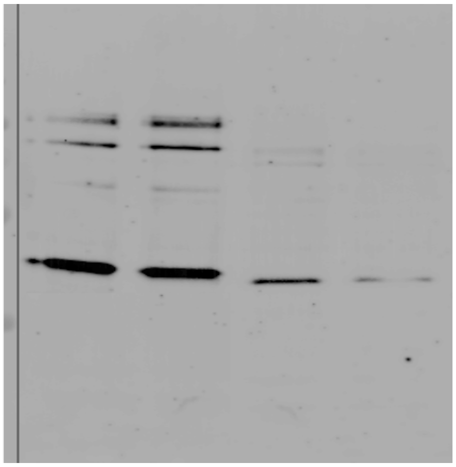

Ecad expression in siRNA treated ML explants

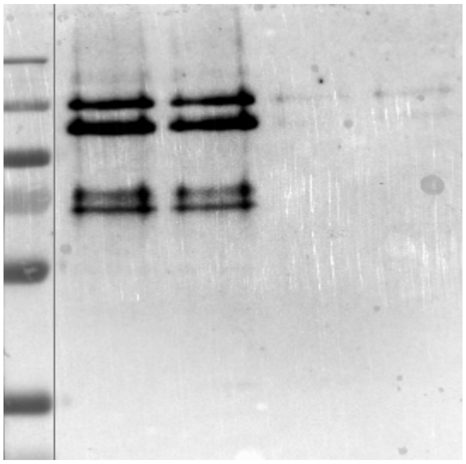

α-SMA expression in siRNA treated ML explants

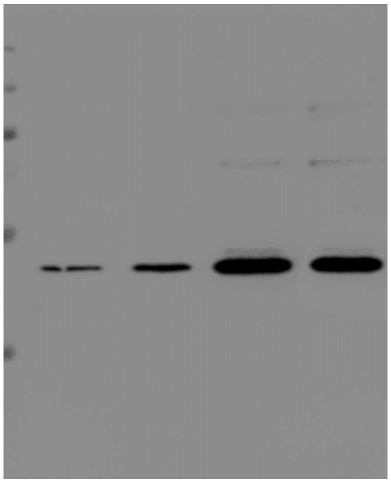

Fibronectin expression in siRNA treated ML explants

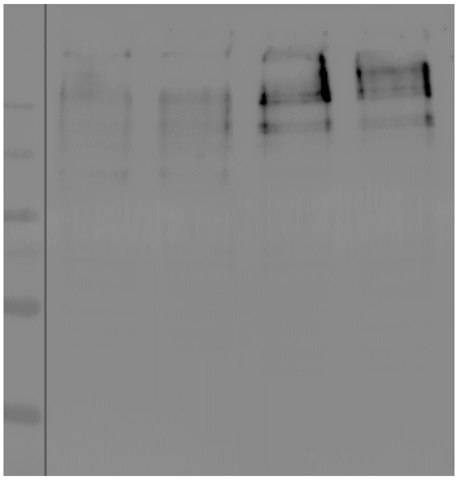

GAPDH expression in siRNA treated ML explants

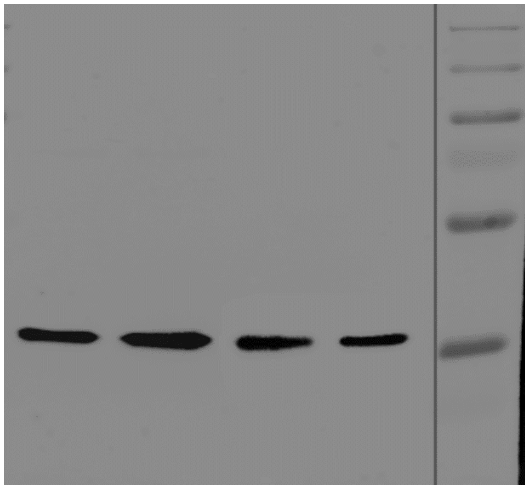

Fig. S1

Total FAK expression in siRNA treated MLEC

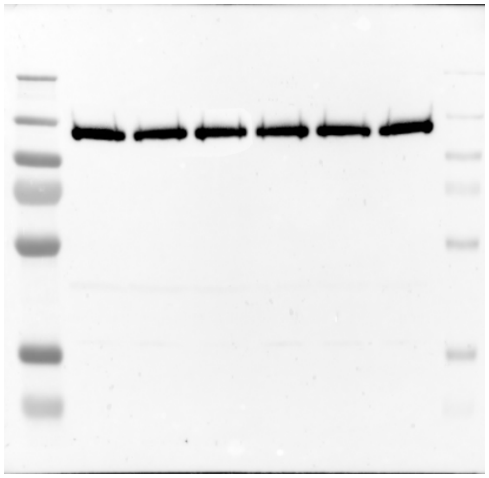

Total Pax expression in siRNA treated MLEC

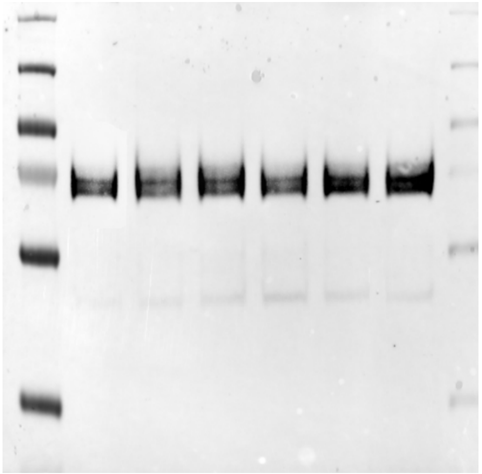

Total MLC expression in siRNA treated MLEC

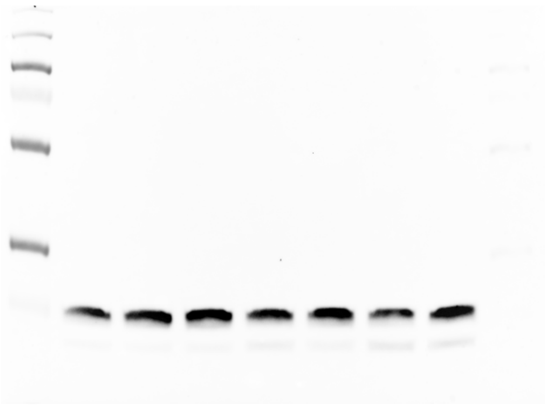

Supplement: Supplementary file 2 — Supplement for original Western blots and Gels. [file 41598_2020_58189_MOESM2_ESM.pdf]
